# Two electrolyte decomposition pathways at nickel-rich cathode surfaces in lithium-ion batteries†

**DOI:** 10.1039/d1ee04053g

**Published:** 2022-07-05

**Authors:** Bernardine L. D. Rinkel, J. Padmanabhan Vivek, Nuria Garcia-Araez, Clare P. Grey

**Affiliations:** Department of Chemistry, University of Cambridge Cambridge CB2 1EW UK cpg27@cam.ac.uk; School of Chemistry, University of Southampton Southampton SO17 1BJ UK; The Faraday Institution, Harwell Campus Didcot OX11 0RA UK

## Abstract

Preventing the decomposition reactions of electrolyte solutions is essential for extending the lifetime of lithium-ion batteries. However, the exact mechanism(s) for electrolyte decomposition at the positive electrode, and particularly the soluble decomposition products that form and initiate further reactions at the negative electrode, are still largely unknown. In this work, a combination of *operando* gas measurements and solution NMR was used to study decomposition reactions of the electrolyte solution at NMC (LiNi_*x*_Mn_*y*_Co_1−*x*−*y*_O_2_) and LCO (LiCoO_2_) electrodes. A partially delithiated LFP (Li_*x*_FePO_4_) counter electrode was used to selectively identify the products formed through processes at the positive electrodes. Based on the detected soluble and gaseous products, two distinct routes with different onset potentials are proposed for the decomposition of the electrolyte solution at NMC electrodes. At low potentials (<80% state-of-charge, SOC), ethylene carbonate (EC) is dehydrogenated to form vinylene carbonate (VC) at the NMC surface, whereas at high potentials (>80% SOC), ^1^O_2_ released from the transition metal oxide chemically oxidises the electrolyte solvent (EC) to form CO_2_, CO and H_2_O. The formation of water *via* this mechanism was confirmed by reacting ^17^O-labelled ^1^O_2_ with EC and characterising the reaction products *via*^1^H and ^17^O NMR spectroscopy. The water that is produced initiates secondary reactions, leading to the formation of the various products identified by NMR spectroscopy. Noticeably fewer decomposition products were detected in NMC/graphite cells compared to NMC/Li_*x*_FePO_4_ cells, which is ascribed to the consumption of water (from the reaction of ^1^O_2_ and EC) at the graphite electrode, preventing secondary decomposition reactions. The insights on electrolyte decomposition mechanisms at the positive electrode, and the consumption of decomposition products at the negative electrode contribute to understanding the origin of capacity loss in NMC/graphite cells, and are hoped to support the development of strategies to mitigate the degradation of NMC-based cells.

## Abstract 2

NMR and *operando* gas measurements show that at low potentials, EC is dehydrogenated to VC, whereas at high potentials, EC is chemically oxidised to CO_2_, CO and H_2_O, where the water that is formed induces secondary decomposition reactions.

## Graphical abstract

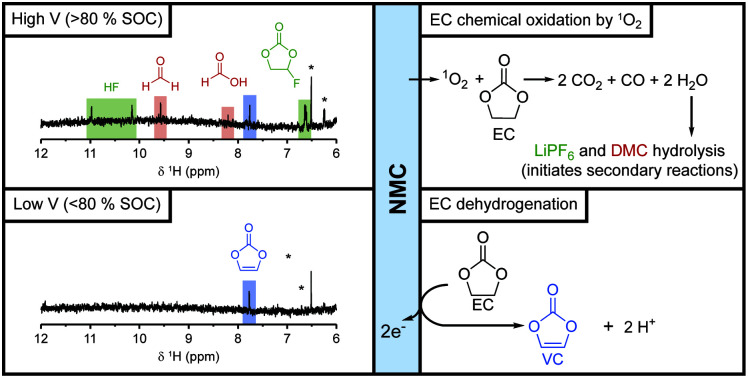

**Broader context**

The development of rechargeable batteries with longer lifetimes represents a major challenge in enabling the shift from fossil fuel-powered to electric vehicles. Ni-rich layered positive electrode materials are now universally used in electric vehicle batteries and yet their degradation pathways are still not understood. One major cause of the loss in capacity of these batteries is linked with the decomposition of the electrolyte solution, and the subsequent reactions of these products at the negative electrode, a process referred to as “electrode cross-talk”. In the present work, we characterise the electrolyte decomposition products formed at LiNi_*x*_Mn_*y*_Co_1−*x*−*y*_O_2_ (NMC) positive electrodes using solution NMR spectroscopy and *operando* gas measurements. We generate ^17^O-labelled singlet oxygen (^1^O_2_) photochemically and use it to investigate proposed electrolyte decomposition pathways. Two main and distinct decomposition mechanisms are identified, each with a different onset voltage, and very different decomposition products. Characterisation of NMC/graphite “full cells” reveals that many of the products formed at the NMC electrode are consumed at the negative electrode, emphasising the importance of studying electrode cross-talk processes to understand the origin of capacity loss in NMC/graphite batteries.

## 1 Introduction

The use of Ni-rich layered transition metal oxides (*e.g.*, LiNi_*x*_Mn_*y*_Co_1−*x*−*y*_O_2_, NMC) as positive electrode materials in lithium-ion battery packs is favoured over LiCoO_2_ due to their higher energy densities, and because cobalt, with its toxicity, cost and mining issues, is largely replaced with nickel.^1–3^ However, batteries using these Ni-rich NMCs and a graphite negative electrode suffer from rapid capacity fade, limiting the lifetime of the battery.^3–5^ This is often ascribed to degradation mechanisms at the positive electrode, including reconstruction of the surface layers and concomitant loss of lattice oxygen,^4,6,7^ electrolyte oxidation,^4,8–11^ transition metal dissolution and deposition on the negative electrode,^12–15^ and cracking of the electrode particles,^16–18^ all leading to an impedance rise and loss of active material at the positive electrode.^19–21^ However, analysis of electrochemical data (incremental capacity analysis, ICA, differential voltage analysis, DVA, and coulombic efficiency measurements) have clearly shown that the dominant source for the capacity loss in NMC and NCA/graphite cells is due to the loss of active lithium inventory due to parasitic reactions that occur at the graphite electrode.^22–27^

The rate of capacity fade increases proportionally with a higher Ni-content in the positive electrode material,^3^ suggesting that even though the capacity loss might be directly due to side reactions at the negative electrode, it may be indirectly or in part due to processes at the positive electrode. In particular, electrolyte decomposition products formed at the positive electrode diffuse to the negative electrode where they are reduced and deposited, in a process called “electrode cross-talk”.^21,26,28–33^ The reduction of those oxidation products at the negative electrode may consume active lithium ions and/or electrons,^30–32^ and the deposition of those products can limit ion transport to the bulk of the negative electrode,^24,26^ both of which can make significant contributions to the capacity fade.

Several mechanisms have been proposed for electrolyte decomposition at the positive electrode, each with a different onset potential and resulting in the formation of different products. Electrochemical (or faradaic) oxidation has been reported to occur at potentials greater than ∼5 V *vs.* Li^+^/Li, resulting in the formation of CO_2_ (measured), acetaldehyde, ethylene oxide and various radical species (predicted by density functional theory (DFT) calculations).^34–40^ Gasteiger and co-workers proposed an alternative route involving the chemical oxidation of the electrolyte solvent by reactive oxygen species. Using emission spectroscopy and on-line electrochemical mass spectrometry (OEMS) measurements, it was revealed that reactive oxygen species (singlet oxygen, ^1^O_2_) are released from the transition metal oxide (TMO) lattice at high states of delithiation (state of charge, SOC, >80%) and it was proposed that these species chemically oxidise the electrolyte, producing CO_2_ and CO gas and water.^4,6,8,9^ The onset potential for this mechanism is material dependent: ∼4.3 V *vs.* Li^+^/Li for NMC811, ∼4.7 V *vs.* Li^+^/Li for NMC622 and NMC111,^4,8^ and it is correlated with surface reconstruction phenomena resulting in the release of lattice oxygen.^41^ Unfortunately, CO_2_ and CO are “generic” reaction products, and – without knowledge of the soluble products that form – do not provide a thorough understanding of how the electrolyte solution is decomposed. Recently, Shao-Horn and co-workers proposed a third pathway based on DFT calculations, where EC is dehydrogenated on the surface of the positive electrode.^42–44^*In situ* Fourier-transform infrared spectroscopy (FT-IR) revealed the formation of VC, dehydrogenated EC and dehydrogenated oligomers on the surface of NMC811 electrodes at potentials as low as 3.8 V *vs.* Li^+^/Li.^45^ However, it remains unclear what decomposition products form, how they are formed, and which electrolyte decomposition pathways are dominant and most detrimental to the cell lifetime. Furthermore, since it is still not clear what decomposition products (particularly the soluble products) are formed at the positive electrode, it is challenging to investigate cross-talk effects and to fully understand what drives the capacity fade in Ni-rich NMC/graphite cells.

This work reveals not one, but two distinct mechanisms for electrolyte decomposition at NMC electrodes, each with a different onset potential. The soluble electrolyte decomposition products formed at LiNi_1/3_Mn_1/3_Co_1/3_O_2_ (NMC111), LiNi_0.5_Mn_0.3_Co_0.2_O_2_ (NMC532), LiNi_0.6_Mn_0.2_Co_0.2_O_2_ (NMC622), LiNi_0.8_Mn_0.1_Co_0.1_O_2_ (NMC811), and LiCoO_2_ (LCO) electrodes are identified for the first time using solution NMR spectroscopy. Partially delithiated LFP (LiFePO_4_) counter electrodes are used to identify the species formed at NMC electrodes, as LFP neither produces or consumes electrolyte decomposition products.^46–51^ Complementary *operando* pressure and OEMS measurements are performed to provide a more complete picture of the various decomposition products that are formed, and the combined NMR and gas results are subsequently used to infer the decomposition reactions that occur. At low potentials (<80% SOC), EC is dehydrogenated to VC, without the release of gaseous decomposition products. At high potentials (*i.e.*, >80% SOC), ^1^O_2_ chemically oxidises EC to produce CO_2_ and CO gas, and H_2_O. The onset potential for this route thus depends on the material's oxygen evolution potential. The water that is formed through the reaction between EC and ^1^O_2_ results in further decomposition of the electrolyte solution and various species that are identified by NMR spectroscopy. The formation of water in this mechanism is confirmed by reacting photochemically-generated ^17^O-labelled ^1^O_2_ with EC and characterising the products *via*^1^H and ^17^O NMR. Finally, the products formed in NMC/graphite cells are compared to those formed at the NMC electrode. Fewer products are observed in the NMC/graphite cells, which is ascribed to the reduction of the water (formed through the reaction between ^1^O_2_ and EC) at the graphite electrode.

## 2 Experimental

### 2.1 Electrodes and electrolytes

For the NMR measurements, NMC111, NMC532, NMC622, NMC811, and LCO electrodes were prepared by grinding the active material powder (Targray), Super P carbon (Timcal), and Kynar polyvinylidene difluoride (PVDF, Arkerma) in an 8 : 1 : 1 mass ratio with an agate mortar and pestle. Graphite electrodes were prepared by mixing the active material (Targray), Super P carbon and polyvinylidene difluoride (PVDF) in a 92 : 2 : 6 ratio. *N*-Methyl-2-pyrrolidone (NMP, 99.5%, anhydrous, Sigma-Aldrich) was added to form a slurry, which was mixed in an agate mortar and pestle and then blade-coated at a wet film thickness of 300 μm on an aluminium (NMCs and LCO) or copper (graphite) foil current collector. The films were dried at 60 °C until most of the solvent was removed and were subsequently dried at 100 °C for 16 hours. The LCO, NMC111 and graphite electrodes were prepared in an ambient atmosphere, whereas the NMC532, NMC622 and NMC811 electrodes were prepared in an argon-filled glovebox (O_2_ and H_2_O < 1 ppm, MBraun), as these Ni-rich active materials react with CO_2_ and H_2_O to form undesirable carbonates and hydroxide surface groups.^52^ Disks of the desired size were cut (active material loading 5–6 mg cm^−2^ for NMCs and LCO, 4.5 mg cm^−2^ for graphite) and dried further at 100 °C under vacuum for 24 hours, before being transferred into an argon-filled glovebox (O_2_ and H_2_O < 1 ppm; MBraun). Commercially sourced LFP electrodes (LiFePO_4_, PI-KEM, 12 mg cm^−2^) were cut to the desired size (n : p ratio ≈ 1.5 for NMC/LFP and LCO/LFP cells) and dried at 100 °C under vacuum for 24 hours, after which they were transferred into an argon-filled glovebox.

For the *operando* pressure measurements and on-line electrochemical mass spectrometry (OEMS) measurements, the electrodes were coated on a fine steel mesh (SS316 grade, The Mesh Company) to allow better gas diffusion from both sides of the electrode. NMC electrodes were prepared by mixing the active material powder (Targray), PVDF (PVDF 5130, Solvay), and Super C65 conductive carbon black (Timcal), in 90 : 5 : 5 mass ratio, and NMP (Sigma-Aldrich, 99.5%, anhydrous) was added to this to form a slurry. Partially delithiated iron phosphate (Li_0.25_FePO_4_; Li_*x*_FP) counter electrodes used for the OEMS studies were prepared by mixing LiFePO_4_, FePO_4_, PVDF and Super C65 carbon in a 22 : 66 : 6 : 6 mass ratio, as described previously.^53^ FePO_4_ was produced by chemical delithiation of LiFePO_4_ following our previous work (see Fig. S1, ESI† for potential profile).^54^ The NMC electrodes had active material loadings of 3–4 mg cm^−2^, and an additional OEMS experiment was done with a higher active material loading of 9 mg cm^−2^. The Li_0.25_FePO_4_ had an active material loading of 15 mg cm^−2^, thus providing a 1.9 higher capacity than the NMC electrode with loading of 3 mg cm^−2^. The inks were mixed in a planetary mixer (Thinky ARE-250) three times at 2000 rpm for 5 minutes, with 5 minute breaks in between for cooling. The slurry was then blade-coated on a fine steel mesh using an automatic film coater (MTI, MSK-AFA-III) to a wet thickness of 200 μm. Prior to coating, the steel mesh was calendared to remove creases; an aluminium foil was placed under the mesh during doctor-blading. The slurry coated mesh was then transferred to a vacuum oven and dried at 80 °C for 12 hours. The electrodes were punched in discs of 25 mm using a handheld precision punch (Nogami, Japan), then pressed using a hydraulic pellet press (Specac) at 5 tonne pressure. The electrodes were further dried for 48 hours in a Buchi glass vacuum oven (6 hours at 25 °C, 8 hours at 80 °C, 12 hours at 100 °C then 22 hours at 120 °C) then the sealed glass oven was transferred to an argon filled glovebox (MBraun, Germany; O_2_ and H_2_O < 1 ppm). In a similar way, Glass Fibre B separator and partially delithiated LFP counter electrodes (where applicable) were also cut to 25 mm discs then dried and transferred to the glovebox. All the Swagelok cell components were dried under vacuum at 80 °C for 12 hours.

For all cells a 1 M LiPF_6_ in ethylene carbonate (EC) and dimethyl carbonate (DMC) electrolyte (LP30; EC : DMC = 50 : 50 (v/v), battery grade, Sigma-Aldrich, or anhydrous, Solvionic) was used, unless otherwise specified. To study the contributions of the EC and DMC solvent separately, electrolyte solutions of 1.5 M LiPF_6_ (99.9%, Solvionic) in EC (battery grade, ≥99%, <10 ppm H_2_O, Sigma-Aldrich) and 1 M LiPF_6_ in DMC (battery grade, ≥99%, <10 ppm H_2_O, Sigma-Aldrich) were prepared. Electrolytes from Soulbrain of 1 M LiPF_6_ in EC or 1 M LiPF_6_ in DMC were also employed. The oxidation of methanol (hydrolysis product of DMC) at NMC and LCO electrodes was investigated by preparing a solution of LP30 + 2% methanol (99.8%, anhydrous, Sigma-Aldrich).

### 2.2 Two-electrode cells

NMC and LCO electrodes were cycled against partially delithiated LFP (Li_*x*_FePO_4_, *x* < 1) or graphite electrodes in a standard coin cell configuration. Cells using partially delithiated LFP as the negative electrode were used to selectively identify the decomposition products formed at the positive electrode (NMC or LCO), as the LFP electrode should not produce or consume electrolyte decomposition products. LFP operates at a potential (∼3.45 V *vs.* Li^+^/Li) that is within the electrochemical stability window of the carbonate-based electrolyte^55–57^ and it does not release reactive species at a high state-of-charge (SOC). Therefore, the electrolyte in the cell should nominally only contain decomposition products originating from the NMC or LCO electrode. Cells using graphite as the negative electrode were prepared to study the electrolyte decomposition products that are formed in a commercial Li-ion battery.

The LFP electrodes were delithiated by assembling LFP/Li cells (lithium metal foil; 99%, Aldrich), which were cycled to the flat region in the discharge curve (for cycling protocol see electrochemical methods). After delithiating the LFP electrode, the cells were opened and the partially delithiated LFP electrodes were washed with DMC (3 × 0.3 mL) and dried under dynamic vacuum for 30 min, to remove any electrolyte decomposition products originating from the lithium metal electrode.

The delithiated LFP electrodes were reassembled into NMC/LFP or LCO/LFP cells with an N/P ratio (the capacity ratio of the negative to the positive electrode) of 1.5, based on the practical capacities of the NMCs, LCO and LFP obtained in lithium half cells. The capacity of the LFP electrodes was oversized so that the electrodes would operate in the flat region (at relatively constant potential) of the LFP charge/discharge curves, as this made it easier to control the upper potential reached by the NMC and LCO electrodes. For the NMC/graphite and LCO/graphite cells a lower N/P ratio of ∼1.2 was chosen, to resemble more closely N/P ratios used in commercial cells.

Two-electrode cells were assembled in a standard coin cell configuration: the positive electrode (NMC or LCO) on a stainless-steel current collector, a borosilicate glass fibre separator (Whatmann, GF/B, 0.68 mm thick, 1.0 μm pore size) wetted with 150 μL of electrolyte solution or a polypropylene separator (Celgard 3501) wetted with 100 μL of electrolyte solution, the negative electrode (delithiated LFP or graphite), on a stainless-steel current collector and cone spring were compressed in a 2032-type coin cell casing. The glass fibre separator was used to act as a sponge for the electrolyte, so that the electrolyte could be easily extracted after disassembling the cell. All cell assembly was performed in an argon-filled glovebox.

### 2.3 Three-electrode cells

To determine the upper potential reached by the NMC and LCO electrodes in the NMC/LFP cells, three-electrode NMC/Li/LFP cells were assembled: A NMC working electrode (WE), partially delithiated LFP counter electrode (CE) and lithium metal reference electrode (RE), were assembled in a Swagelok cell with 200 μL of electrolyte solution.

### 2.4 Electrochemical methods

Electrochemical measurements were performed at 25 °C using a Biologic MPG2 potentiostat/galvanostat instrument running EC-lab software. The LFP electrodes were delithiated by charging the LFP/Li cells to 4.0 V_cell_, followed by a 24 hour potential hold to ensure complete delithiation, after which the cells were discharged to 3.6 V to partially (re)lithiate the electrode. Charging and discharging was performed in constant current (CC) mode at a C/5 rate (based on a practical capacity of 127 mA h g^−1^).

The (NMC or LCO)/LFP cells were cycled between 0.2 and 0.73, 0.93, 1.13, 1.33 or 1.53 V_cell_, corresponding to an upper potential of 4.1, 4.3, 4.5, 4.7 and 4.9 V *vs.* Li^+^/Li for the positive electrode. The (NMC or LCO)/graphite cells were cycled between 2.5 and 4.65 V_cell_, corresponding to an upper potential of *ca.* 4.7 V *vs.* Li^+^/Li for the positive electrode. Charging was performed at C/5, either in constant current-constant voltage (CCCV) mode with a current limitation corresponding to 0.02 C or in CC mode with a 2 hour potential hold at the upper voltage limit. Discharging was performed in CC mode at a C/5 rate. Cells charged in CCCV mode were cycled 20 times, whereas cells charged in CC mode were cycled 10 times. Practical capacity values of 160, 167, 175 and 200 mA h g^−1^ were used for NMC's 111, 532, 622 and 811, respectively, for the C-rate calculations.

NMC/Li/delithiated LFP three-electrode cells were cycled twice between 0.2 and 1.53 V_cell_ at a C/5 rate in CC mode with a 2 hour potential hold.

### 2.5 *Operando* pressure measurements & on-line electrochemical mass spectrometry (OEMS)

**Fig. 1:**
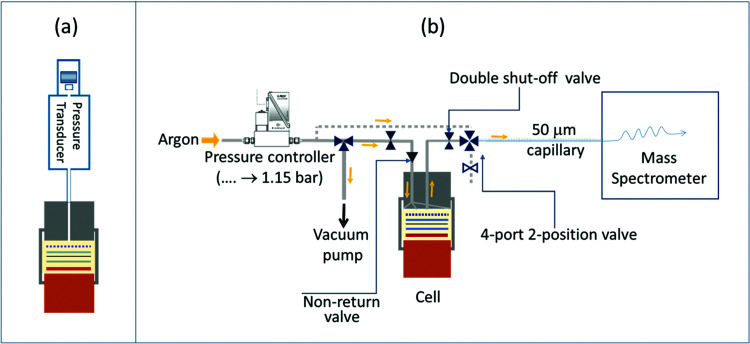
Schematic of the *operando* pressure cell (a) and the on-line electrochemical mass spectrometry (OEMS) setup (b).

Gas evolution for different NMC cathodes (NMC111, NMC622, NMC532 and NMC811) was studied by monitoring the internal pressure changes of a Swagelok-type cell during cycling. A pressure transducer (PA-33X, Keller Druck AG) attached to a standard 1 inch Swagelok cell, was used to monitor the internal pressure of the cell (Fig. 1a). The cell was designed with a small headspace volume (2.55 mL), which provided high sensitivity in the detection of gas formation.^58^ As in a typical Swagelok cell, a copper current collector was used for the lithium metal counter electrode and a steel current collector was used for the NMC electrode in the NMC/Li cells; an aluminium current collector was used when partially delithiated LFP was used as the counter electrode. The cell was assembled inside an argon-filled glovebox (O_2_ and H_2_O < 1 ppm) as follows: a 25 mm lithium foil disc was placed on the copper current collector at the base of the cell then 200 μL of electrolyte solution was added to the centre of the lithium metal disc, then a GF/B separator was placed on top of this and another 200 μL of electrolyte solution was added to the centre of the separator, then the NMC disc electrode was placed on top of this ensuring proper alignment of the electrodes and the separator. The steel current collector was then placed on top of the NMC electrode and the sealed cell was brought outside of the glovebox and further tightened to ensure sealing; it was then transferred to a climatic chamber set to 25 °C. After a 24 hour rest at 3 V (OCV) to allow the temperature and pressure to stabilize, the cell was cycled between 3.0 V and a series of upper cut-off potentials: 4.3, 4.4, 4.5, 4.55, 4.6, 4.65, 4.7, 4.75 and 4.8 V. For each upper cut-off potential, the cell was cycled twice in CC mode: once without and once with a 2 hour potential hold at the top of charge.

OEMS experiments were performed to determine which gases are produced at high potentials and give rise to the increased internal cell pressure. For these measurements only NMC811 cathodes were employed, as it has previously been shown that the nature of the gases is the same for various NMCs and only the onset potential for gas evolution differs.^8^ NMC811 was chosen, as the *operando* pressure measurements showed that this material produced the most gaseous products. After a 4 hour rest at OCV, the cell was charged to 4.3, 4.5 and 4.7 V and held at each potential for 2 hours, before going to the next target potential. Charging was performed in CC mode at a C/5 rate.

The OEMS setup consisted of a quadrupole mass spectrometer (Pfeiffer Thermostar) connected to a specially designed electrochemical cell and a 50 μm capillary of the mass spectrometer was connected to the electrochemical cell *via* a manual GC sampling valve (Valco, Fig. 1b). A Swagelok-style electrochemical cell with an inlet and outlet drilled through the cathode current collector was used for OEMS studies. The outlet of the electrochemical cell was connected to the mass spectrometer capillary *via* the GC sampling valve. The inlet of the electrochemical cell was connected to a pressure controller (EL-Press, Bronkhorst) that fed argon to the cells when the pressure inside the electrochemical cells dropped below 1.15 bar. Between the inlet on the electrochemical cell and the pressure controller, a 3-way valve (Swagelok) connecting to a vacuum pump, allowed contaminant-free transfer of the gases from the electrochemical cell to the OEMS gas line. The outlet of the electrochemical cell had a quick disconnect double shut-off valve assembly (Beswick Engineering, USA) connected to the GC sampling valve, and any dead volume of air trapped in between the internal and external valve assembly was purged out by flowing argon through the outlet valve of the GC sampling adapter. The 50 μm-diameter capillary connected to the mass spectrometer and the capillary inlet were heated to 120 °C to prevent solvent condensation. This design of the OEMS system minimises argon gas flow through the electrochemical cell (*ca.* 9 μL min^−1^) and, thus, minimises solvent evaporation. The cell headspace volume (including the connection to the mass spectrometer) is *ca.* 3 mL. For quantification of the gas evolution rates, the setup was calibrated for H_2_, C_2_H_4_, O_2_, CO and CO_2_ (*m*/*z* values of 2, 26, 32, 28 and 44, respectively) using standard calibration gases of known concentrations (SIP Analytical). Two calibration gas cylinders, one containing H_2_, C_2_H_4_, O_2_, and CO_2_ (each 1000 ppm in Ar) and another one containing 1000 ppm CO and H_2_ in Ar were used separately to avoid overlap of the fragments, following previous work.^30^ Background effects were corrected by fitting a baseline to the signals recorded during the cell rest period, as detailed in previous studies.^10,59^

### 2.6 Singlet oxygen experiments

The chemical reactions between singlet oxygen (^1^O_2_) and the electrolyte solvent were studied by generating ^1^O_2_ in a carbonate solution and analysing the reaction mixture by solution NMR. To produce ^1^O_2_ in an organic carbonate solution, a similar approach was taken to that reported by Freiberg *et al*.:^11^^1^O_2_ was generated in triplet oxygen (^3^O_2_) saturated solutions of ethylene carbonate (EC), dimethyl carbonate (DMC) and vinylene carbonate (VC), by photoexcitation of ^3^O_2_ to ^1^O_2_ using Rose Bengal as a photosensitiser. Irradiating Rose Bengal with light at 525 nm results in the formation of a triplet state of Rose Bengal, which can then transfer energy to ^3^O_2_ during a collision between Rose Bengal and ^3^O_2_, exciting ^3^O_2_ to ^1^O_2_.^60^

Rose Bengal salt (disodium salt, >95%, Sigma-Aldrich) was dried at 130 °C under dynamic vacuum for 72 hours. Solutions of 100 μM Rose Bengal in EC (battery grade, ≥99%, <10 ppm H_2_O, Sigma-Aldrich), DMC (battery grade, ≥99%, <10 ppm H_2_O, Sigma-Aldrich) and VC (99.9%, Solvionic) were prepared in an argon-filled glovebox. A pre-dried glass vial (7 mL volume) was filled with 3 mL of the Rose Bengal/carbonate solution and a magnetic stir bar was added for convective mixing during irradiation of the solution. The glass vial was fitted with a septum (Suba-Seal), to create an air and moisture tight container, and transferred out of the glovebox. Oxygen gas (>99.9999%, ALPHAGAZ 2, Air Liquide) was bubbled through the solution for 10 minutes to ensure the Rose Bengal/carbonate solution was saturated with oxygen. For experiments using ^17^O-enriched O_2_ (70% enriched, CortecNet), the solution was first saturated with “regular” oxygen (99.76% ^16^O_2_, 0.2% ^18^O_2_ and 0.04% ^17^O_2_), before bubbling through the ^17^O_2_.

The glass vial containing the Rose Bengal/carbonate solution was placed on a magnetic stirrer between four LEDs and irradiated with light at 525 nm. Each LED had a maximum emission between 520–530 nm and a luminous flux of 120 lumens at a maximum power of 3 W, operating at a voltage between 3.4–4 V and a current of 750 mA. The LEDS are connected in series and are mounted on an aluminium casing that acts as a heat sink for heat generated during the experiment (Fig. S2, ESI†). A current of 400 mA was applied to the LEDs for 2 hours, after which 0.1 mL of the Rose Bengal/carbonate solution was taken for analysis by solution NMR.

### 2.7 Solution NMR

After cycling the (NMC or LCO)/LFP and (NMC or LCO)/graphite cells, the cells were disassembled in an argon-filled glovebox and the glass fibre separator was soaked in 0.7 mL of DMSO-d_6_ (Aldrich, 99.9 atom% D, 99% CP) for 5 minutes. The solution was transferred to an airtight NMR tube fitted with a Young's tap. ^1^H NMR spectra of each batch of DMSO-d_6_ used for this work were also acquired (Fig. S8, ESI†) to identify which signals arise from impurities in the deuterated solvent.

One-dimensional ^1^H, ^19^F{^1^H} and ^17^O NMR spectra and two-dimensional ^1^H–^1^H correlation spectroscopy (COSY) NMR spectra were recorded on a Bruker AVANCE III HD 11.7 T (*ω*_1H_ = 500 MHz) spectrometer using a BBO probe. ^1^H spectra were internally referenced to DMSO-d_6_ at 2.50 ppm (*δ*^1^H) and ^19^F and spectra were internally referenced to LiPF_6_ at −74.5 ppm (*δ*^19^F). The ^17^O NMR spectra were externally referenced to D_2_O at 0 ppm.

## 3 Results

### 3.1 Analysis of gaseous electrolyte decomposition products in NMC/Li cells by *operando* pressure measurements and OEMS

#### 3.1.1 *Operando* pressure measurements

The onset potential for gas evolution at the positive electrode was first determined by cycling the different NMC/Li half-cells for the same cycling protocol, while measuring the internal pressure of the cell. After a 24 hour potential hold at 3 V to obtain a baseline, the cells were cycled twice between 3.0 V and a series of increasing upper cut-off potentials (4.3–4.85 V), where the upper potential was increased in steps of 0.1 V up to 4.5 V, after which the step size was reduced to 0.05 V. The cells were then cycled twice to 4.3 V again to observe the cell pressure after cycling to high cell potentials. For every cut-off potential, the second cycle included a 2 hour hold at the top of charge to allow enough time for the electrolyte solution to be decomposed and any associated changes in pressure, due to gas evolution, to stabilise and be accurately measured. A lithium metal counter electrode was chosen, since it has already been shown to provide a stable reference potential.^4,58^

**Fig. 2:**
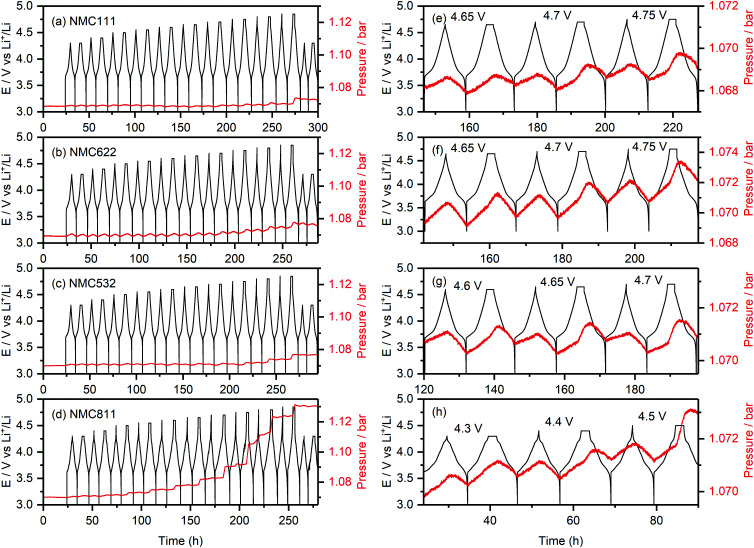
*Operando* pressure data for (a) NMC111, (b) NMC622, (c) NMC532 and (d) NMC811/Li cells using an LP30 electrolyte. The cells were cycled between 3.0 V and a series of increasing upper cut-off potentials (4.3–4.85 V), after which they were cycled to 4.3 V again; the potential was stepped by 0.1 V every two cycles until 4.5 V, whereafter the step-size was reduced to 0.05 V. For every cut-off potential value, the second cycle included a 2 hour potential hold at the top of charge. The internal cell pressure and potential-time data are shown in red and black, respectively. An expanded view of the data is shown on the right (e–h). The active material mass loading was 3 mg cm^−2^ for all cells and all measurements were performed at 25 °C.

Fig. 2 shows the internal cell pressure overlaid with the cell potential as a function of time for NMC111, NMC622, NMC532 and NMC811/Li half-cells with a 1 M LiPF_6_ in EC : DMC (1 : 1, v/v; LP30) electrolyte. The cell pressures stay constant during the 24 hour hold at 3 V (*vs.* Li^+^/Li) prior to cycling, and then increase and decrease periodically (1.0–1.5 mbar) as the cells were charged and discharged. These cyclic fluctuations in the cell pressure are due to volumetric changes of the positive and negative electrodes during lithiation and delithiation, and are dominated by changes in the lithium metal electrode volume (*i.e.*, the plating and stripping of lithium).^58,61^ For NMC811 (Fig. 2h), the overall cell pressure increases slowly during the first two cycles between 4.3 and 3.0 V (by about ∼0.2 mbar), but starts to rise noticeably during the 2 hour hold at 4.4 V *vs.* Li^+^/Li (0.5 mbar), and keeps increasing as the cell was cycled to higher upper cut-off potentials. For the other NMC compositions, a small increase in cell pressure was seen up to 4.65 V (∼0.2 mbar). However, more pronounced rises in pressure were observed during the 2 hour potential hold for NMC532 at 4.65 V *vs.* Li^+^/Li (Fig. 2f), and NMC622 and NMC111 at 4.7 V *vs.* Li^+^/Li (Fig. 2g and e). For the final two cycles to 4.3 V, the cell pressures remain constant in all the cells, demonstrating high stability and absence of measurement artefacts (*e.g.*, leaks).

The pronounced increase in cell pressure at high potentials (4.4 V *vs.* Li^+^/Li for NMC811, 4.65 V *vs.* Li^+^/Li for NMC532, and 4.7 V *vs.* Li^+^/Li for NMC622 and NMC111) is ascribed to the evolution of gaseous decomposition products from electrolyte decomposition at the positive electrode.^4,8,10,62^ These gas evolution onset potentials are consistent with those previously reported for NMC811 (∼4.3 V *vs.* Li^+^/Li), and NMC11 and NMC622 (∼4.6–4.7 V *vs.* Li^+^/Li),^4,8,10^ and correspond to a state-of-charge (SOC) of ∼80% (*i.e.* the material is 80% delithiated) for each of the NMCs (Table 1).

**Table 1:** Onset potential for gas evolution at NMC electrodes as determined by *operando* pressure measurements, and the corresponding state-of-charge (SOC) of the material. SOC values are obtained using the theoretical capacity of the materials (277.3, 276.6, 277.7 and 275.6 mA h g^−1^ for NMC111, NMC622, NMC532 and NMC811, respectively) and assuming 100% Coulombic efficiency. The values for LiCoO_2_ (LCO) were taken from our previous work^63^

Material	Gas evolution onset potential (V *vs.* Li^+^/Li)	SOC (%)
NMC111	4.7	84
NMC622	4.7	84
NMC532	4.65	80
NMC811	4.4	83
LCO	4.6	83

The evolution of gaseous products was predominantly detected during the 2 hour potential hold (in the second cycle at each cut-off potential), showing that the extent of electrolyte decomposition is directly related to the time the cell spends at the top of charge. This is confirmed by NMR analysis of the amount decomposition products formed in NMC811/LFP cells cycled with a CCCV protocol compared to cells cycled in CC mode followed by a 2 hour potential hold (see Fig. S9, ESI† and Fig. 4). The small increase in cell pressure at low voltages (∼0.2 mbar) is attributed to the evolution of gaseous decomposition products from the reduction of the electrolyte solution at the lithium metal negative electrode: immediately when the cell is assembled, the electrolyte solution is reduced at the lithium metal surface, releasing gaseous products and creating the layer of insoluble reduction products on top of the electrode surface known as the solid electrolyte interphase (SEI). While this layer, once formed, self-limits further electrolyte decomposition, the plating and stripping of lithium during cycling may crack the SEI and expose fresh surfaces for continued electrolyte reduction and gas formation. This contribution to the cell pressure is expected to increase proportionally to the amount of lithium extracted from the cathode and, thus, cannot explain the more pronounced increase in cell pressure at higher voltages.

To understand which of the electrolyte components (EC, DMC or LiPF_6_) is decomposed and gives rise to the increased cell pressure, the *operando* pressure measurements were repeated for NMC811/Li cells, using DMC-only (1 M LiPF_6_ in DMC) and EC-only (1.5 M LiPF_6_ in EC) electrolytes. For pure EC electrolyte solutions, a higher electrolyte salt concentration was required to prevent the solution from solidifying at ambient temperatures. The gas evolution onset potential for the cell using the DMC-only electrolyte was at 4.7 V (Fig. S3, ESI†), indicating that the gaseous products formed at lower potentials do not originate from DMC decomposition. For the cell with the EC-only electrolyte, the gas evolution onset potential (4.4–4.5 V, Fig. S4, ESI†) matches that of the cell using the combined EC/DMC (1 : 1 v/v) electrolyte (4.4 V), showing that the gaseous products formed at this voltage result from the EC solvent.

#### 3.1.2 Electrochemical mass spectrometry (OEMS) measurements

**Fig. 3:**
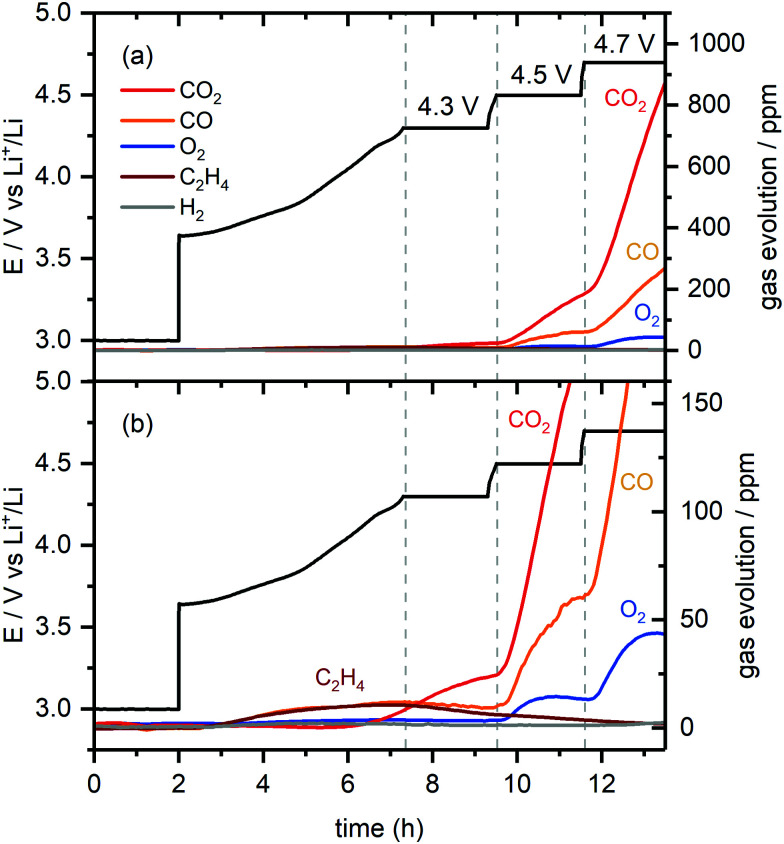
(a) Gas evolution in a NMC811/Li cell charged from 3.0 to 4.7 V (*vs.* Li^+^/Li) with a 2 hour potential hold at 4.3, 4.5 and 4.7 V as measured by online-electrochemical mass spectrometry (OEMS). The cell potential-time data is given in black. The gas concentration in the cell head space are given in ppm for CO_2_ (*m*/*z* = 44), CO (*m*/*z* = 28), O_2_ (*m*/*z* = 32), C_2_H_4_ (*m*/*z* = 26) and H_2_ (*m*/*z* = 2). (b) Enlarged view of the gases evolved at low concentrations (between 0–700 ppm). The active material mass loading was 4 mg cm^−2^ and the measurement was performed at 25 °C.

The results of on-line electrochemical mass spectrometry (OEMS) measurements on NMC811/Li cells charged from 3.0 V to 4.3, 4.5 and 4.7 V *vs.* Li^+^/Li at a C/5 rate and held at each cell potential for 2 hours, while detecting the evolution of CO_2_, CO, O_2_, C_2_H_4_ and H_2_ gas, are shown in Fig. 3. During the initial potential hold at 3.0 V, the gas signals remain constant, indicating that no gaseous electrolyte decomposition products are being formed. Below the gas evolution onset potential identified from the *operando* pressure measurements (4.4 V *vs.* Li^+^/Li for NMC811), small amounts of CO, CO_2_ and C_2_H_4_ are evolved: the CO signal increases almost linearly, whereas the formation of C_2_H_4_ reaches a maximum at 4.3 V. The CO_2_ signal increases linearly from ∼4.0 V until the end of the 2 hour hold at 4.3 V. After charging the cell to 4.5 V (above the gas evolution onset potential at 4.4 V), a sharp increase of the CO_2_ and CO signals was observed, as well as the evolution of O_2_. The O_2_ signal plateaus during the 2 hour hold period, whereas the CO_2_ and CO signals (3 : 1 ratio) continue to rise. As the cell is charged to 4.7 V, the O_2_ signal increases initially and plateaus again, while the CO_2_ and CO signals (4 : 1 ratio) increase even more steeply. The H_2_ signal remained constant throughout the experiment, implying that the amount of H_2_ generation was negligible.

**Fig. 4:**
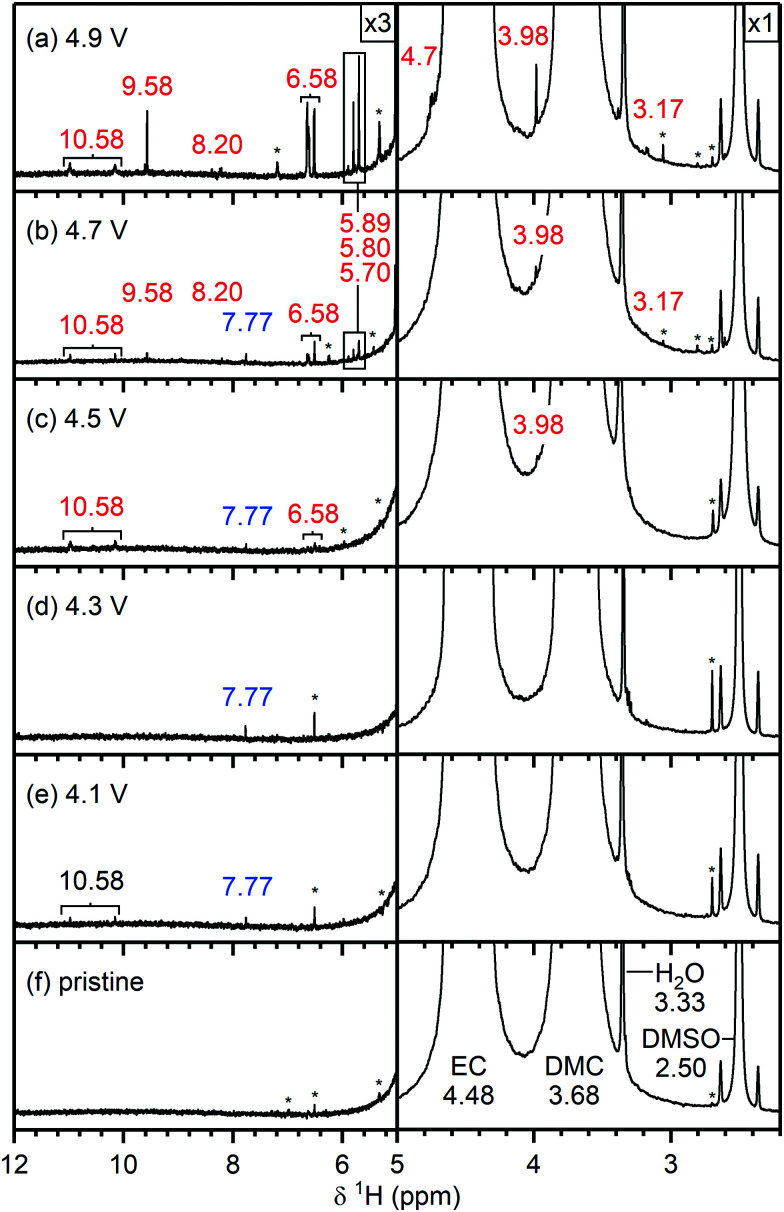
^1^H NMR spectra of electrolyte solutions extracted from NMC811/LFP cells after 10 cycles, where the cell cut-off voltages were chosen so the NMC electrode was cycled between (a) 4.9 V, (b) 4.7 V, (c) 4.5 V, (d) 4.3 V, (e) 4.1 V and 3.0 V *vs.* Li^+^/Li, and (f) pristine electrolyte solution. The cells were cycled at rate of C/5 in constant current (CC) mode with a 2 hour potential hold at the top of charge. The region between 5–12 ppm is enlarged 3 times compared to the region between 3–5 ppm. The signals of ethylene carbonate (EC; 4.48 ppm), dimethyl carbonate (DMC; 3.68 ppm) and dimethyl sulfoxide (DMSO; 2.50 ppm) are annotated in black on the bottom spectra. The chemical shifts (in ppm) correspond to signals that appeared below (blue) and above (red) the gas evolution onset potential (4.4 V *vs.* Li^+^/Li for NMC811). The cell cycled to 4.1 V (e) contained a weak signal for HF (10.58 ppm, black), as a slightly older electrolyte solution was used in this cell. The asterisks denote impurities in the deuterated DMSO solvent. The NMC active material mass loading was 4–5 mg cm^−2^.

The formation of C_2_H_4_ and CO at low cell potentials, <4.3 V *vs.* Li^+^/Li, is ascribed to processes at the lithium metal electrode, as reported previously:^10,30,64^ the evolution of C_2_H_4_ and CO at low potentials, arises from EC and DMC reduction,^64–66^ while CO_2_ evolution is ascribed to acid–base reactions between acidic species in the electrolyte solution (*e.g.*, HF) and carbonate species on the surface of the NMC electrode (*e.g.*, Li_2_CO_3_), as well as the slow hydrolysis of the organic carbonate electrolyte.^10,67,68^ At 4.3 V, most (∼75%) of the capacity of the NMC electrode has been reached, and thus, at the lithium counter electrode, most of the lithium has already been plated, which explains why the rate of C_2_H_4_ production decreases. To confirm that this C_2_H_4_ and CO evolution originates from processes at the lithium metal electrode, the OEMS measurement was repeated using Li_0.25_FePO_4_ as a counter electrode. The mass loading of the Li_0.25_FePO_4_ electrode was increased to ensure that the potential of the Li_*x*_FePO_4_ remains in the constant potential plateau region during cycling. No C_2_H_4_ and CO gas were evolved at low cell potentials in this cell (Fig. S5, ESI†), confirming that these gases are formed at the lithium metal electrode.

The small amount of CO_2_ evolved at the potential hold at 4.3 V suggests that the reaction of ^1^O_2_ release that triggers EC decomposition is slow, but not negligible, at this lower potential. Additional experiments were done with cells with higher NMC loadings, which produced the same gaseous products but in a higher concentration, thus confirming that all the gases detected are formed due to the electrode reactions with the electrolyte solution (Fig. S6, ESI†).

### 3.2 Analysis of soluble electrolyte decomposition products formed at NMC electrodes by solution NMR spectroscopy

To identify any soluble electrolyte decomposition products formed at the positive electrode, NMC electrodes were cycled to below and above the gas evolution onset potential as determined by the *operando* pressure measurements. As above, partially delithiated Li_1−*x*_FePO_4_ (LFP) was used as the counter electrode to prevent decomposition of the electrolyte solution at the counter electrode. NMC811, NMC622, NMC532 and NMC111/LFP cells were cycled to a cell voltage of 0.73, 0.93, 1.13, 1.33 and 1.53 V, corresponding to 4.1, 4.3, 4.5, 4.7 and 4.9 V *vs.* Li^+^/Li as confirmed by using a three-electrode cell (Fig. S7, ESI†). After cycling, the NMC/LFP cells were disassembled, and the glass fibre separators were soaked in deuterated DMSO to extract the electrolyte solution and its soluble decomposition products for analysis by solution NMR.

The ^1^H and ^19^F NMR signals observed for the NMC811, NMC622, NMC532 and NMC111 cells are similar; the species that are formed are identical, only the signal intensity and the potential at which the signals appear vary for the different NMCs. For the following analysis, the NMR spectra from the NMC811/LFP cells are shown, as these samples had the highest signal intensities of the decomposition products. The ^1^H and ^19^F NMR spectra for the NMC622, NMC532, NMC111 and LCO cells are shown in the ESI† (Fig. S14–S21). A list of the species observed in the electrolyte solutions, the potential at which they were observed and their chemical shifts, is given in Table 2 and summarised below.

**Table 2:** Summary of the electrolyte solution decomposition products formed at the NMC811 electrode as identified by solution NMR and the potential (*vs.* Li^+^/Li) at which they were first detected

Potential at which first observed (*vs.* Li^+^/Li)	Species	Nucleus	Chemical shift (ppm)
Pristine electrolyte	Ethylene carbonate (EC)	^1^H	4.48 (s)
	Dimethyl carbonate (DMC)	^1^H	3.68 (s)
	Lithium hexafluorophosphate (LiPF_6_)	^19^F	−74.5 (d, ^1^*J*_P–F_ = 710 Hz)
	OPF_2_(OH)	^19^F	−83.1 (d, ^1^*J*_P–F_ = 955 Hz)

4.1 V	Vinylene carbonate (VC)	^1^H	7.77 (s)

4.5 V	HF	^1^H	10.58 (d, ^1^*J*_F–H_ = 410 Hz)
		^19^F	−171.7 ppm (s)
	Lithium fluoroborate (LiBF_4_)	^19^F	−152.7 (s)
	Silicon fluorides (SiF_*x*_)	^19^F	−138.89(s)
	Fluoroethylene carbonate (FEC)	^1^H	6.58 (ddd, ^2^*J*_F–H_ = 60.7 Hz; ^3^*J*_H–H_ = 4.1, 0.7 Hz);
			4.73 (ddd, ^2^*J*_F–H_ = 36.3 Hz; ^3^*J*_H–H_ = 11.0, 4.2 Hz);
			4.64 (ddd, ^2^*J*_F–H_ = 21.3 Hz; ^3^*J*_H–H_ = 11.0, 0.7 Hz)
		^19^F	−126.5 (s)

4.7 V	Formaldehyde	^1^H	9.58 (s)
	Formic acid	^1^H	8.20 (s)
	Acetal; C***H̲***_2_(OR)_2_; methanediol (R = H), methoxymethanol (R^1^ = OCH_3_, R^2^ = H)	^1^H	5.80 (s)
			5.70 (s)
	Methanol	^1^H	4.10 (q, ^3^*J*_H–H_ = 5.5 Hz)
			3.17 (d, ^3^*J*_H–H_ = 5.5 Hz)
	OPF_2_(OCH_3_)	^1^H	3.98 (s)

#### 3.2.1 ^1^H NMR

Fig. 4 shows the ^1^H NMR spectra of the pristine electrolyte solution (bottom) and the electrolyte solutions extracted from NMC811/LFP cells cycled to 4.1, 4.3, 4.5, 4.7 and 4.9 V *vs.* Li^+^/Li.

##### Pristine electrolyte solution

The ^1^H NMR spectrum of the pristine electrolyte solution (Fig. 4f) shows two main signals arising from the two co-solvents: ethylene carbonate (EC; 4.48 ppm) and dimethyl carbonate (DMC; 3.68 ppm).^69,70^ Traces of water were detected by the appearance of a singlet at 3.33 ppm,^71^ however no hydrofluoric acid (doublet at ∼10.6 ppm)^72,73^ was observed. The presence of HF in the pristine electrolyte solution depends on the age of the solution and the level of water contamination and may also arise from batch-to-batch variations. The signal at 2.50 ppm is assigned to non-deuterated DMSO impurities in the DMSO-d_6_ solvent.^71^ Unfortunately the impurities vary between different batches of deuterated DMSO and thus the signals assigned to DMSO impurities (marked with an asterisk) will differ between the spectra (see Fig. S8, ESI†).

##### Electrolyte solution from cycled NMC811/LFP cells

After cycling to 4.1 and 4.3 V, the ^1^H NMR spectra of the electrolyte solutions (Fig. 4e and d) show the presence of vinylene carbonate (VC; 7.77 ppm). A small signal for HF (10.58 ppm) is present in the cell cycled to 4.1 V (Fig. 4e) as a slightly older electrolyte solution was used in this cell. For the cell cycled between 4.5–3.0 V (Fig. 4c), the electrolyte solution now also contains HF (10.58 ppm), fluoroethylene carbonate (FEC; 6.62, 4.73, 4.64 ppm) and the fluorophosphate ester OPF_2_(OCH_3_) (3.98 ppm), in addition to VC. For the cells cycled to 4.7 and 4.9 V (Fig. 4a and b), several new signals appear between 10^−3^ ppm in the ^1^H NMR spectrum: formaldehyde (9.58 ppm), formic acid (8.20 ppm), acetals (methoxymethanol and methanediol; 5.80 and 5.70 ppm) and methanol (3.17 ppm). The signal for VC has disappeared in the cell cycled between 4.9–3.0 V. While the ^1^H NMR signals are weak, the total amount of decomposition product formed after 10 cycles between 4.7–3.0 V (Fig. 4b) corresponds to ∼1000 ppm (quantified by NMR integration). The measurements were repeated for cells assembled with a polypropylene separator and the results were similar to those obtained when a glass fibre separator was used (Fig. S12, ESI†).

No signs of transition metal dissolution were seen: no bulk magnetic susceptibility effects (*i.e.*, change in chemical shift) or broadening of the NMR signals was observed, indicating that the concentration of dissolved ions is low. Moreover, the electrolyte solutions were diluted with DMSO, which minimises any potential effects arising from dissolved transition metal ions. Studies with much longer term cycling have revealed BMS effects/shifts and broadening of the main resonances, but a more detailed study is required to correlate this with the concentration and nature of the dissolved paramagnetic (transition) metal ions.

#### 3.2.2 ^19^F NMR

##### Pristine electrolyte solution

**Fig. 5:**
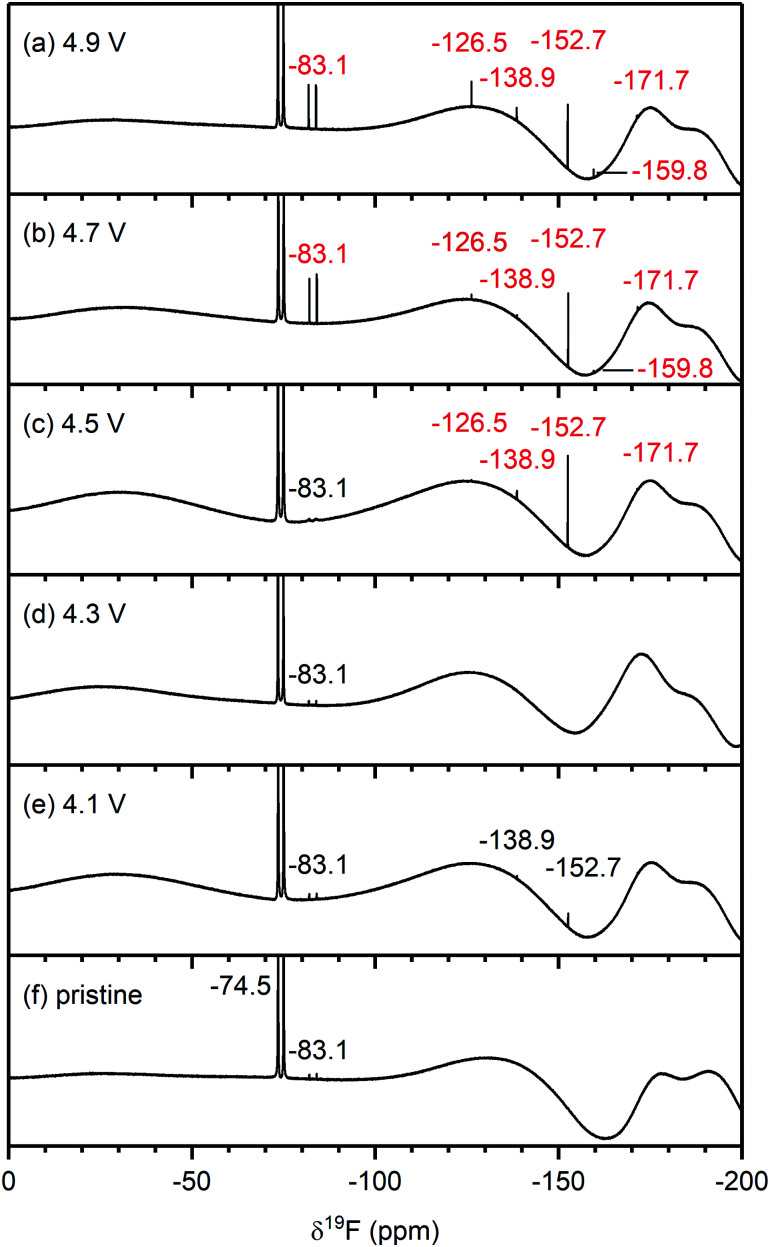
^19^F NMR spectra of electrolyte solutions extracted from NMC811/LFP cells after 10 cycles, where the cell cut-off voltages were chosen so the NMC electrode was cycled to (a) 4.9 V, (b) 4.7 V, (c) 4.5 V, (d) 4.3 V, (e) 4.1 V and 3.0 V *vs.* Li^+^/Li, and (f) pristine electrolyte solution. The cells were cycled at rate of C/5 in constant current (CC) mode with a 2 hour potential hold at the top of charge. The chemical shifts of the electrolyte salt (LiPF_6_, −74.5 ppm), LiPF_6_ hydrolysis products commonly found in the pristine electrolyte (PO_2_F_2_^−^, −83.1 ppm) and species that arise from HF impurities in the pristine electrolyte (SiF_*x*_,−138.9 ppm and BF_4_^−^, −152.7 ppm) are given in black. The chemical shifts in blue and red correspond to signals that appeared below and above the gas evolution onset potential (4.4 V *vs.* Li^+^/Li for NMC811), respectively. For (c), a weak signal for FEC appears at −126.5 ppm, (see also Fig. S11, ESI†). The broad features at approximately −130 ppm and −175 and −190 ppm arise from the NMR probe and are not due to additional species. The NMC active material mass loading was 4–5 mg cm^−2^.

The ^19^F{^1^H} spectrum of the pristine electrolyte solution (Fig. 5f) shows a large signal from LiPF_6_ (−74.5 ppm, d, ^1^*J*_P–F_ = 710 Hz) and a minor signal from LiPO_2_F_2_/OPF_2_OH (−83.1 ppm, d, ^1^*J*_P–F_ = 955 Hz) impurities.^70^

##### Electrolyte solution from cycled NMC811/LFP cells

The ^19^F NMR spectra of electrolyte solutions from cells cycled up to 4.3 V (Fig. 5d and e) reveal only small quantities of LiPO_2_F_2_, comparable to those found in the pristine electrolyte. The cell cycled between 4.1–3.0 V also contains lithium fluoroborate (LiBF_4_/BF_4_^−^; −152.7 ppm), which results from the attack on the glass separator by the small HF impurity in the electrolyte solution used in this cell. These signals were not observed when a polypropylene separator was used (Fig. S13, ESI†). After cycling between 4.5–3.0 V (Fig. 5c), the electrolyte contains LiBF_4_/BF_4_^−^ as well as silicon fluoride species (SiF_*x*_, *x* = 4–6, −138.9 ppm). A small signal for FEC (−126.5 ppm) could also be detected, as seen more clearly in the enlarged version of the ^19^F NMR spectrum (Fig. S11, ESI†). For the cells cycled to well above the gas evolution onset potential (∼4.4 V for NMC811), the ^19^F NMR spectra (Fig. 5a and b) reveal increased concentrations of fluorophosphate esters and FEC, and the presence of and HF (−171.7 ppm),^72,73^ in addition to the boron and silicon fluoride species and that were observed at 4.5 V.

To summarise, for NMC/LFP and LCO/LFP cells cycled to below the positive electrode gas evolution onset potential, the only soluble decomposition product detected in the electrolyte solution is VC. For cells cycled above the evolution potential, a number of species are formed: formaldehyde, formic acid, FEC, acetals, methanol, HF, LiBF_4_, SiF_*x*_ and OPF_2_(OCH_3_). These species were observed for all NMCs (NMC811, NMC622, NMC532 and NMC11) and LCO, and the potential at which they appeared is correlated to the respective gas evolution onset potentials of each electrode. As the LFP electrode does not decompose the electrolyte solution, the observed species are assumed to have formed through processes occurring at the NMC (and LCO) electrodes.

### 3.3 Analysis of soluble decomposition products formed in NMC/graphite cells by solution NMR

To understand if the electrolyte decomposition products formed at the NMC electrode (as discussed above) react further or are consumed at the graphite electrode, the electrolytes from cycled NMC811/graphite cells were compared to those of the NMC/LFP cells. For the following analysis, the electrolytes from NMC811-based cells are shown, and again similar results were obtained for the other NMC compositions and LCO (see Fig. S22 and S23, ESI† for the ^1^H and ^19^F NMR spectra, respectively). The electrolyte from an LFP/graphite cell was also analysed to identify the electrolyte decomposition products originating from the graphite electrode, so these signals would not be incorrectly assigned to decomposition products from the NMC electrode.

**Fig. 6:**
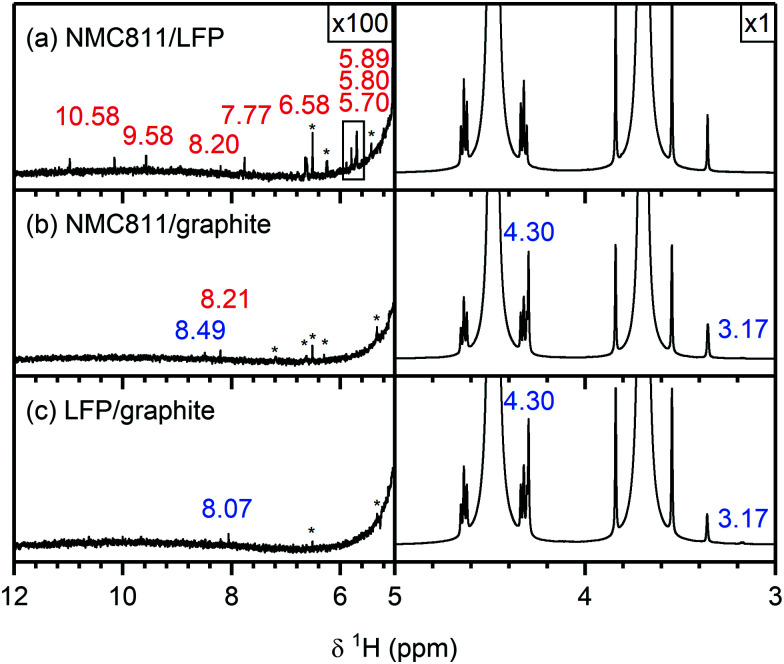
^1^H NMR spectra of electrolyte solutions extracted from (a) NMC811/LFP, (b) NMC811/graphite and (c) LFP/graphite cells after 10 cycles. The cells were cycled between 1.13–0.2 V_cell_, 4.65–2.5 V_cell_ and 3.45–2 V_cell_, respectively, corresponding to upper potential values for NMC and graphite of 4.7 V and 0.05 V *vs.* Li^+^/Li, respectively, at rate of C/5 in constant current (CC) mode with a 2 hour potential hold at the top of charge. The region between 5–12 ppm is enlarged 100 times compared to the region between 3–5 ppm. The signals arising from species formed at the NMC and graphite electrode are annotated in red and blue, respectively. The NMC active material mass loading was 4–5 mg cm^−2^.

**Fig. 7:**
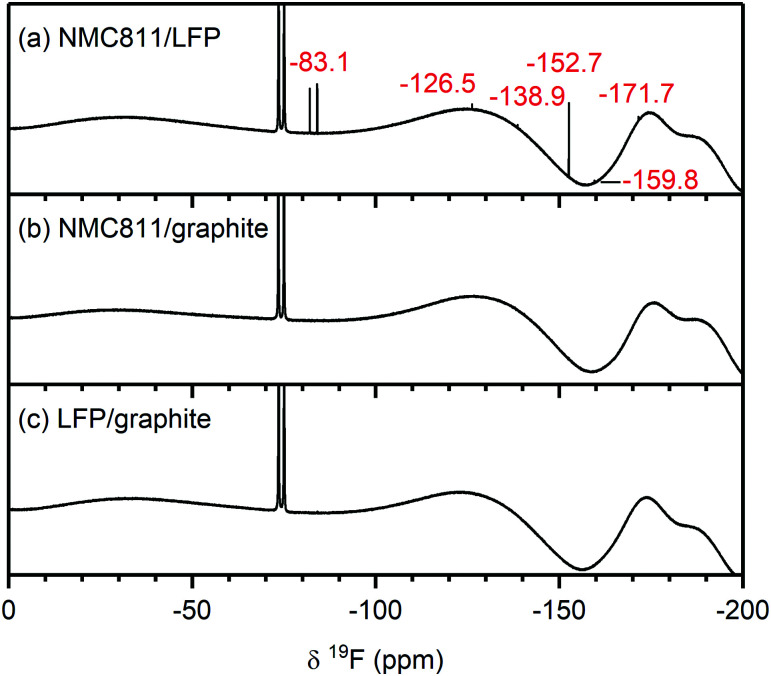
^19^F NMR spectra of electrolyte solutions extracted from (a) NMC811/LFP, (b) NMC811/graphite and (c) LFP/graphite cells after 10 cycles. The cells were cycled between 1.13–0.2 V_cell_, 4.65–2.5 V_cell_ and 3.45–2 V_cell_, respectively, corresponding to a *V*_NMC_ = 4.7 V and a *V*_graphite_ = 0.05 V *vs.* Li^+^/Li, at rate of C/5 in constant current (CC) mode with a 2 hour potential hold at the top of charge. The signals arising from species formed at the NMC and graphite electrode are annotated in red. The broad features at approximately −130 ppm and −175 and −190 ppm arise from the NMR probe and are not due to additional species. The NMC active material mass loading was 4–5 mg cm^−2^.

The ^1^H NMR spectra of the electrolytes from cycled NMC811/LFP (*V*_cell_ = 1.33 V, *V*_NMC_ = 4.7 V *vs.* Li^+^/Li), NMC811/graphite (*V*_cell_ = 4.65 V, *V*_NMC_ = 4.7 V *vs.* Li^+^/Li) and LFP/graphite cells are shown in Fig. 6. The species formed at the NMC811 electrode when cycled to 4.7 V *vs.* Li^+^/Li have already been discussed above (in NMC811/LFP cells). On the other hand, the results from graphite/LFP cells show that the major decomposition species formed at the graphite electrode are lithium ethylene dicarbonate (LEDC; 4.30 ppm) and methanol (4.10, 3.18 ppm, Fig. 6c, right). The only two signals observed between 12–5 ppm are assigned to formic acid/formate (tentatively; 8.07 ppm) and a DMSO impurity at 6.53 ppm (Fig. 6c, left). Interestingly, the ^1^H NMR spectrum of the electrolyte from the NMC811/graphite cell shows fewer signals than that of the NMC811/LFP cell (Fig. 6b): a small signal for formic acid remains and a new signal for lithium formate (8.49 ppm) appears. However, the signals for HF, formaldehyde, VC and acetals are not present in the ^1^H NMR spectrum of the NMC811/graphite cell. Finally, the ^19^F NMR spectra of the NMC811/LFP cell (Fig. 7a) shows the presence of PO_2_F_2_^−^, FEC, SiF_*x*_, BF_*x*_ and HF (as discussed above), while no fluorine-containing decomposition products are observed in the electrolyte from both the NMC811/graphite and the LFP/graphite cells (Fig. 7b and c).

### 3.4 Reaction between singlet oxygen and the electrolyte solvent

To determine whether any of the electrolyte decomposition products identified in the NMC/LFP cells are formed through the reaction of singlet oxygen (^1^O_2_) with the carbonate solvent of the electrolyte solution, ^1^O_2_ was produced in a carbonate solution and the reaction mixture was analysed by solution NMR. Rose Bengal was used as a photosensitiser to generate ^1^O_2_ in triplet oxygen (^3^O_2_)-saturated solutions of ethylene carbonate (EC), vinylene carbonate (VC) and dimethyl carbonate (DMC), by irradiating the solution with light at 525 nm. First, to confirm that ^1^O_2_ was indeed produced by the photosensitiser, a solution of dimethyl anthracene (DMA) and Rose Bengal in EC was irradiated for 2 hours, and analysis by solution NMR indicated the formation of the endo-peroxide of DMA, confirming that ^1^O_2_ was generated (Fig. S24, ESI†).^74^

**Fig. 8:**
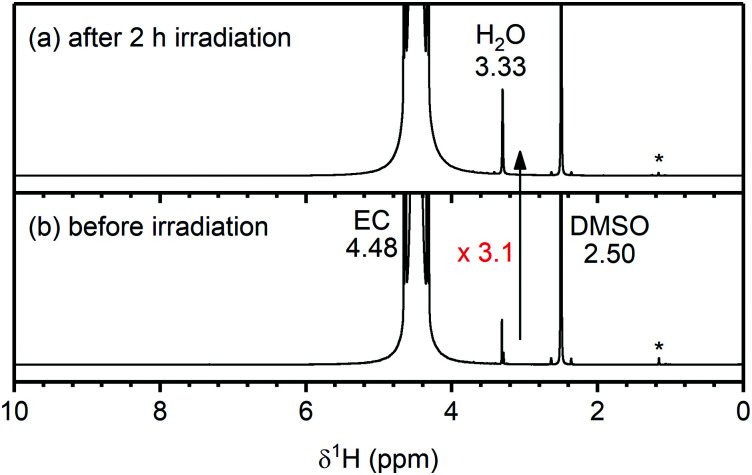
^1^H NMR spectra of an ethylene carbonate (EC) and Rose Bengal (100 μM) solution (a) after and (b) before 2 hours of irradiation at 525 nm to generate singlet oxygen (^1^O_2_). The chemical shifts of EC, H_2_O and DMSO are annotated in black. An enlarged spectrum is given in Fig. S25 (ESI†). After generation of ^1^O_2_ in solution, the H_2_O signal increased 3.1 times.

The ^1^H NMR spectra of the Rose Bengal/EC solution before and after irradiation for 2 hours are shown in Fig. 8. Before irradiation, signals from EC (4.48 ppm), water (3.33 ppm) and non-deuterated DMSO (2.50 ppm) are detected, as well as DMSO impurities at (1.2–1.1 ppm). A small signal from Rose Bengal is observed at 7.32 ppm (see enlarged spectrum in the ESI,† Fig. S25). After irradiating the solution for 2 hours, the water signal increased by a factor of 3.1 (determined by integration and corrected for volume of solution used for each spectrum) and the Rose Bengal signal shifted to 6.52 ppm. No other additional signals were observed and in particular, no signals for VC (7.77 ppm) or H_2_O_2_ (10.27 ppm)^75,76^ were seen (Fig. 8a and Fig. S25, ESI†). VC and H_2_O_2_ have previously been proposed as the reaction products for the chemical oxidation of EC by ^1^O_2_.^11^

To determine whether the water observed after irradiation was produced from a reaction between EC and ^1^O_2_ (and did not originate from water adsorbed to glassware, for example), ^17^O-enriched O_2_ gas was used to selectively label the reaction products of ^1^O_2_, so they could be identified by ^17^O NMR. If the water originated from a non-enriched source (*i.e.*, adsorbed to glassware), the ^17^O signal for water should increase by a similar amount as the ^1^H signal (∼3×), however, if the water originated from a reaction involving ^17^O-enriched ^1^O_2_, the ^17^O water signal may increase by more than a factor of 3.

**Fig. 9:**
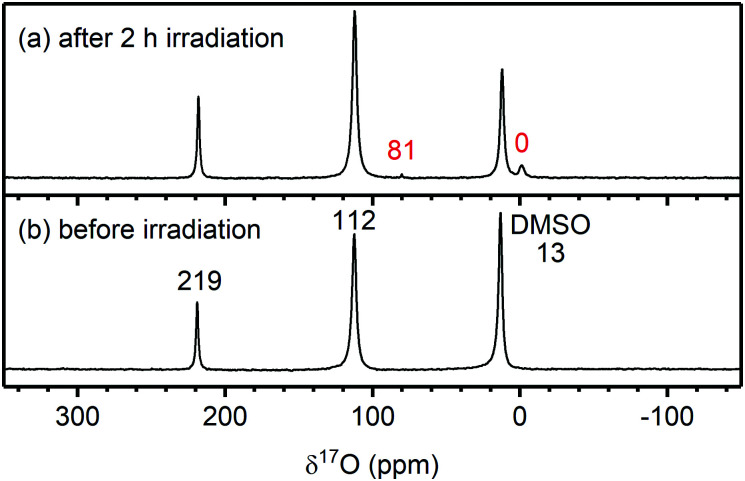
^17^O NMR spectra of an ethylene carbonate (EC) and Rose Bengal 100 μM solution (a) after and (b) before 2 hours of irradiation at 525 nm to generate ^17^O-enriched singlet oxygen (^1^O_2_). The chemical shifts of EC (219 and 212 ppm) and DMSO (13 ppm) are annotated in black. The chemical shifts in red correspond to the signals for water (0 ppm) and dissolved CO_2_ (tentatively, 81 ppm), the signals appearing after ^1^O_2_ production in solution.

The ^17^O NMR spectra of the Rose Bengal/EC solution before and after irradiation are shown in Fig. 9. Before irradiation, the NMR spectrum shows signals for the carbonyl oxygen of EC (C=***O̲***; 219 ppm), the ***O̲***–(C=O)–***O̲*** oxygens of EC (112 ppm) and DMSO (13 ppm), all originating from unenriched solvent molecules. No water signal could be detected due to the low concentration of water in the electrolyte combined with the low natural abundance of ^17^O (∼0.04%).^77^ After irradiation, two weak signals appear at 81 and 0 ppm. The latter is assigned to ^17^O-labelled water based on a reference sample, and the former is tentatively assigned to dissolved ^17^O-labelled CO_2_ (C^16^O^17^O).^78^ As the ^17^O NMR signal of water increased by at least a factor of 5, it must originate from the ^17^O-enriched ^1^O_2_ (without ^17^O-enrichment of the ^1^O_2_, no new ^17^O NMR signals appeared; Fig. S26, ESI†). This indicates that the reaction between ^1^O_2_ and EC produces water (as its only soluble product). Based on signal integration and taking into account the different levels of ^17^O-enrichment, ∼100 ppm of water was formed during irradiation, *via* a mechanism discussed below.

As discussed above, previous work has suggested that the reaction between EC and ^1^O_2_ results in the formation of VC and H_2_O_2_,^11^ however, neither species could be detected in the solution by ^1^H NMR after reacting EC with ^1^O_2_ in the present work (Fig. 8a and Fig. S25, ESI†). This implies that either VC does not form or, as previously suggested, VC rapidly reacts with ^1^O_2_. To confirm if VC rapidly reacts with ^1^O_2_, ^1^O_2_ was generated in Rose Bengal/VC solutions and the products were again characterised by solution NMR.

The ^1^H NMR spectrum of the solution before irradiation shows a signal for VC (7.77 ppm), and minor signals for impurities from VC (Fig. S27b, ESI†). After irradiation, several new signals appear (Fig. S27a, ESI†): two broad signals are tentatively assigned to poly VC (6.34 and 5.38 ppm),^79,80^ but the main sharp signals that appear at 9.69, 9.11 and 6.46 ppm and are still unassigned. As neither VC or any of the VC + ^1^O_2_ reaction products are present in the ^1^H NMR spectrum of Rose Bengal/EC after irradiation, it is concluded that VC is not a reaction product from the reaction between EC and ^1^O_2_. This also indicates that the formation of VC at NMC electrodes (Fig. 4) does not involve ^1^O_2_. The absence of a signal for H_2_O_2_ cannot be attributed to H_2_O_2_ rapidly decomposing to H_2_O, as the lifetime of H_2_O_2_ in EC is long enough for a signal from H_2_O_2_ to be observed (Fig. S28, ESI†).

Similar experiments were performed with DMC, however, no additional signals nor an increase in the water signal were observed after irradiation (Fig. S29, ESI†). This suggests that DMC does not react with ^1^O_2_, at least under the conditions used here, as has previously been reported.^11^

### 3.5 Oxidation of methanol at the positive electrode surface

To establish whether some of the electrolyte decomposition products (formaldehyde and formic acid) identified in the NMC/LFP cells were formed through the oxidation of methanol (formed through DMC hydrolysis, see discussion), NMC/LFP cells were cycled with an electrolyte containing 2 vol% methanol. Even though methanol has only been observed in cells cycled to 4.7 V and higher, NMC811/LFP cells were cycled to cell voltages of 0.73, 0.93 and 1.13 V, corresponding to 4.1, 4.3 and 4.7 V *vs.* Li^+^/Li, to determine whether methanol could also be oxidised at lower potentials (below the gas evolution onset potential), and thus if the presence of methanol oxidation products can be used as an indicator for the formation of methanol.

**Fig. 10:**
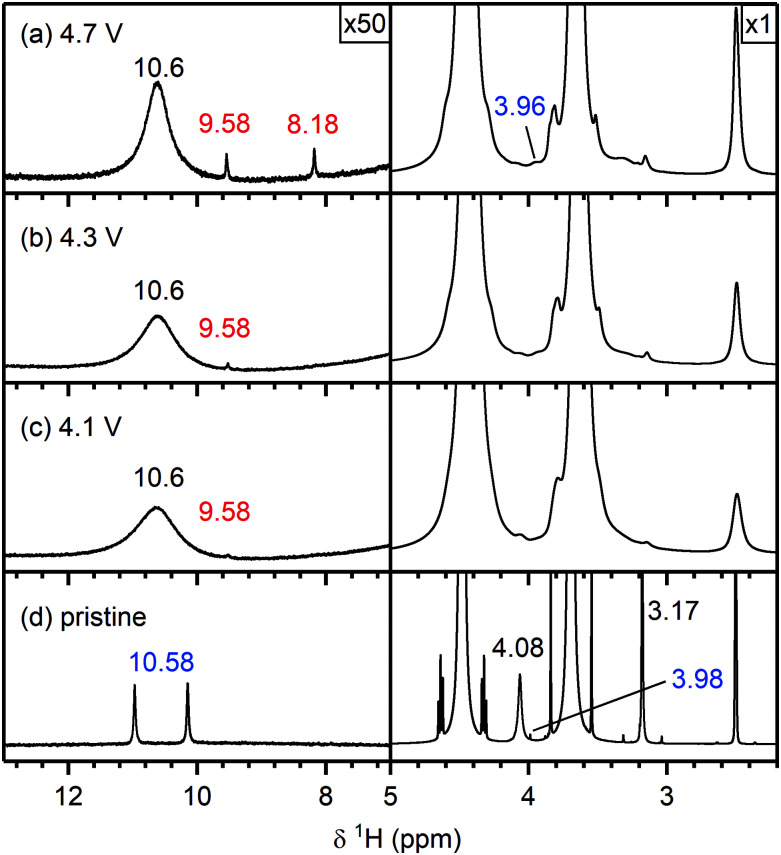
^1^H NMR spectra of electrolyte solutions extracted from NMC811/LFP cells cycled with a 1 M LiPF_6_ in EC : DMC (1 : 1 v/v) + 2 vol% methanol electrolyte solution. The cell upper cut-off voltages were chosen so the NMC electrode was cycled between (a) 4.7 V, (b) 4.3 V, (c) 4.1 and 3.0 V *vs.* Li^+^/Li, and the cells were cycled 10 times at rate of C/5 in constant current (CC) mode with a 2 hour potential hold at the top of charge. (d) The ^1^H NMR spectrum of the pristine electrolyte solution. The region between 5–12 ppm is enlarged 50 times compared to the region between 3–5 ppm. The signals of methanol (4.08 and 3.17 ppm) are annotated in black, the signals arising from the reaction of methanol with the electrolyte solution are shown in blue (HF at 10.58 ppm and OPF_2_(OCH_3_) at 3.98 ppm). The chemical shifts of the signals that appeared after electrochemical cycling are given in red.

Fig. 10 shows the ^1^H NMR spectra of the pristine electrolyte solution (LP30 + 2 vol% methanol) and the electrolyte solutions extracted from the cycled NMC811/LFP cells. The ^1^H NMR spectrum of the pristine electrolyte solution (Fig. 10d) shows signals for methanol (4.08 and 3.17 ppm), a small signal tentatively assigned to the fluorophosphate ester OPF_2_(OCH_3_) (3.98 ppm) and a doublet attributed to HF (10.58 ppm). After cycling the NMC/LFP cells, the ^1^H NMR signals have broadened, most likely as a result of an increased transition metal concentration in the electrolyte due to the high HF concentration.^14,81–83^ For the cells cycled below the gas evolution onset potential (4.1 and 4.3 V), a weak signal assigned to formaldehyde (9.58 ppm) appears (Fig. 10b and c). After cycling between 4.7–3.0 V, the formaldehyde concentration increases and formic acid (8.18 ppm) is observed (Fig. 10a). This indicates that methanol can be oxidised to formaldehyde (at 4.1 V) and formic acid (at 4.7 V) at the transition metal oxide surface.

## 4 Discussion

The results in this work are now used to propose two distinct routes with different onset potentials for electrolyte solution decomposition at the positive electrode. These mechanisms are based on the soluble electrolyte decomposition products identified by solution NMR spectroscopy, the gaseous decomposition products detected by OEMS and a review of the numerous proposed reaction mechanisms in the literature. Based on the identical products that were observed for the various NMC compositions and LCO, it is proposed that these reaction routes will occur for all this class of layered transition metal oxides, but with different onset potentials.

**Fig. 11:**
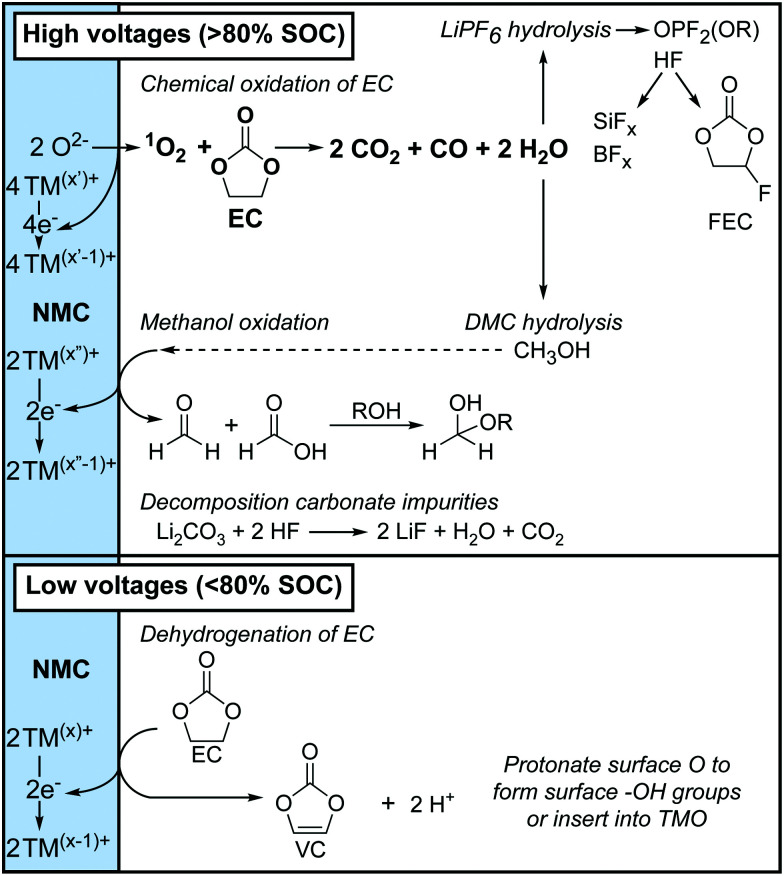
Overview of the electrolyte decomposition reactions that occur at the positive electrode at high potentials (state-of-charge, SOC > 80%) and low potentials (SOC < 80%). The formal oxidation states of the transition metal ions (TM) are indicated with superscripts (*x*, *x*′ and *x*′′) and are illustrative.

The two routes for electrolyte decomposition initiated at the positive electrode are summarised in Fig. 11. In short, at low potentials (*i.e.*, <80% SOC), EC is dehydrogenated to VC, without the formation of gaseous decomposition products. At high potentials (*i.e.*, >80% SOC), ^1^O_2_ is released from the positive electrode and chemically oxidises EC to produce H_2_O, CO_2_ and CO gas, consistent with work by Gasteiger and co-workers.^4,6,8,11^ The water that is formed then hydrolyses both the electrolyte salt and solvent, producing the various species identified by NMR.

### 4.1 Decomposition of the electrolyte solution at low potentials

**Scheme 1:**
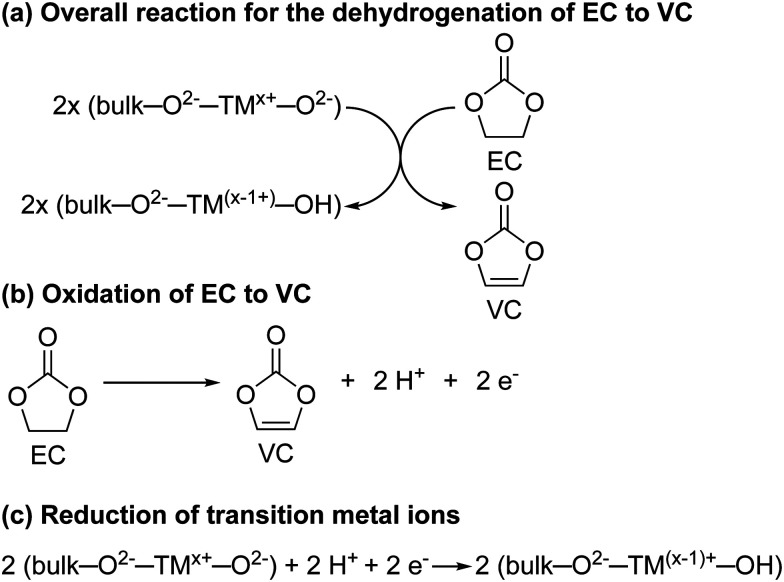
(a) The proposed reaction for the dehydrogenation of ethylene carbonate (EC) to vinylene carbonate (VC) at the surface of the NMC electrode. (b) The electrochemical oxidation of EC to VC. (c) The proposed surface changes at the NMC electrode surface induced by the dehydrogenation of EC to VC. – are used to denote the bond between the transition metal ion (TM) and the oxide/hydroxide groups. The formal oxidation states of the transition metal ions and the oxide/hydroxide group are indicated with superscripts and are illustrative. However, we note that VC is formed at potentials where the formal oxidation state of Ni is only 3+.

For NMC811/LFP cells where the NMC electrodes were cycled between 4.1 and 4.3–3.0 V (*i.e.* below the gas evolution onset potential at 4.4 V *vs.* Li^+^/Li), the only soluble decomposition product in the electrolyte solution detected by solution NMR was VC. The formation of VC at NMC811 electrodes has previously been detected by *in situ* FT-IR measurement at potentials as low as 3.8 V *vs.* Li^+^/Li,^45^ and VC being proposed to form *via* a dehydrogenation mechanism at the electrode surface based on density functional theory (DFT) results.^42,43^ In its simplest form, the dehydrogenation of EC to VC can be written as EC → VC + 2H^+^ + 2e^−^ (Scheme 1b), which is proposed to be driven by the reduction of transition metal ions at the surface of the positive electrode (Scheme 1a) when this reaction occurs chemically; this coupling lowers the overall Δ*G* of the reaction so that it occurs at lower potentials than EC dehydrogenation on *e.g.*, Pt electrodes.^45^ This is supported by the absence of VC formation in cells using Al_2_O_3_-coated NMC electrodes, where direct contact and thus the transfer of electrons between the electrolyte solution and the transition metal oxide active material is blocked, as reported by Shao-Horn and co-workers.^45^

Even though this mechanism results in the formation of protons, none were detected in the electrolyte solution (Fig. 4, no broad signal at ∼14–0 ppm for hydrogen bonded protons or HF), nor do they seem to be associated with other decomposition products (no other decomposition products were detected). One explanation for this is the transformation of oxide surface ions to hydroxide ions (Scheme 1c). A similar, but much more extensive, surface transformation of NMC and LiCoO_2_ materials has been reported upon exposure to water, producing the formation of a transition metal oxy-hydroxide (TMOOH) coating.^84–86^ Another possible fate for the protons generated in the reaction of EC dehydrogenation to VC, is the intercalation of protons in the NMC structure.^87,88^ That this reaction appears self-limiting, *i.e.*, less VC is produced than for example the total Ni^3+^ content of the bulk, suggests that not all O^2−^–Ni^3+^–O^2−^ sites promote this reaction (possibly because the first step involves EC coordination to the surface as suggested by DFT studies)^42,45^ and that the Ni^2+^ and H^+^ produced in the reaction help to passivate the surface. Finally, these results also show that the absence of gas evolution does not indicate that no electrolyte breakdown is occurring.

### 4.2 Decomposition of the electrolyte solution at high potentials

Notably more electrolyte decomposition occurs when the NMC electrodes are polarised to higher potentials. The main gaseous electrolyte decomposition products were determined to be CO_2_ and CO, which evolved in approximately a 3 : 1 or 4 : 1 ratio, and concurrent O_2_ evolution was also detected. The soluble decomposition products found in cells cycled to above the gas evolution onset potential were identified as HF, LiBF_4_, SiF_*x*_, FEC, OPF_2_(OR), formaldehyde, formic acid, acetals and methanol, in addition to VC, which was already present at lower potentials.

#### 4.2.1 Evolution of gaseous electrolyte decomposition products

**Scheme 2:**
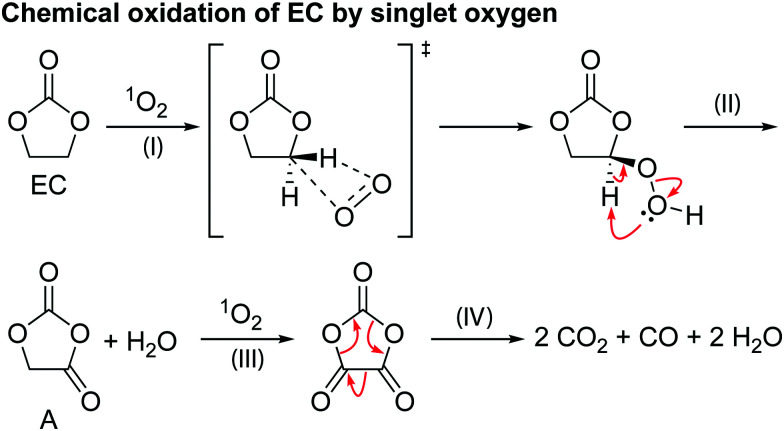
The proposed mechanism for the chemical oxidation of EC by singlet oxygen (^1^O_2_).

The gas evolution onset potentials for the various NMCs were determined to be at 4.4 V (*vs.* Li^+^/Li) for NMC811, 4.65 V (*vs.* Li^+^/Li) for NMC532, and 4.7 V (*vs.* Li^+^/Li) for NMC622 and NMC111, corresponding to a SOC of ∼80% (*i.e.* the material is 80% delithiated) for each of the NMCs. Previous emission spectroscopy experiments have shown that when layered transition metal oxides are charged to 80% SOC, oxygen is released from the lattice as singlet oxygen (^1^O_2_).^6^ Further studies using OEMS measurements revealed that the evolution of CO_2_, CO and O_2_ occurs at the same SOC, and thus it was proposed that the gases formed due to the release of ^1^O_2_ that chemically oxidises the electrolyte solvent to CO_2_ and CO gas and water.^4,6,8,11^ Note that oxygen loss and oxidation of the electrolyte likely occur simultaneously and ^1^O_2_ is not necessarily evolved as a discrete product. The results in this work also support the hypothesis of the formation of gases from the reaction between ^1^O_2_ and the carbonate solvent (Scheme 2), which will be discussed in more detail below.

An additional source of CO_2_ evolution is the decomposition of carbonate residues (*e.g.*, Li_2_CO_3_) present in the NMC active material,^4,10,49,89,90^*via* an electrochemical oxidation (2Li_2_CO_3_ → 2CO_2_ + 4e^−^ + 4Li^+^ + ^1^O_2_)^91^ or *via* an acid–base reaction (*e.g.*, Li_2_CO_3_ + 2HF → 2LiF + H_2_O + CO_2_), where the acidic species (*e.g.*, HF) are formed *via* decomposition reactions of EC triggered by ^1^O_2_.^10,35^ This additional route of CO_2_ formation would explain the ratio of CO_2_ and CO detected here, in which more CO_2_ is evolved than the expected ratio of 2 : 1 for the reaction of EC with ^1^O_2_ (Scheme 2). As will be shown below, the reaction of EC with ^1^O_2_ forms H_2_O, which in turn, induces the decomposition of the LiPF_6_ salt producing HF.

Alternative proposed routes for EC and DMC oxidation proceed *via* an electrochemical (faradaic) reaction.^35–38,92,93^ However, the suggested onset potentials for these mechanisms (∼5.0 V) and the resulting gaseous products (CO_2_ only) are different from the results obtained in this work, thus an electrochemical oxidation mechanism does not explain the electrolyte decomposition products observed in the NMC/LFP cells.

#### 4.2.2 Reaction between ^1^O_2_ and EC

To confirm that ^1^O_2_ causes the decomposition of the electrolyte solution at high potentials and to determine which decomposition products form through this reaction, ^17^O-labelled ^1^O_2_ was generated in a solution of EC and the reaction products were characterised by ^1^H and ^17^O NMR. The main soluble decomposition product in the solution was identified as water (Fig. 8 and 9), demonstrating that ^1^O_2_ does react with the carbonate solvent, and that the generation of water in the electrolyte solution is a signature of this reaction.

Scheme 2 shows a proposed reaction mechanism for the reaction between EC and ^1^O_2_, based on the known reactions of ^1^O_2_^94^ and previously reported mechanisms for the chemical oxidation of EC,^4^ and is consistent with the species identified in the ^1^H and ^17^O NMR spectra. Electrophilic attack of singlet oxygen on the CH_2_ carbon *via* a direct insertion mechanism leads to the formation of a hydroperoxyl group *via* a direct insertion mechanism (step I). The unstable hydroperoxyl group decomposes to form a carbonyl group (intermediate A) and releases water (as detected by NMR, step II). The decomposition of this intermediate A to CO_2_, CO and formaldehyde was dismissed by Gasteiger and co-workers,^4^ as they did not observe the presence of formaldehyde, in agreement with the present findings (note that formaldehyde is only observed in our work at very high potentials, >4.7 V *vs.* Li^+^/Li, due to methanol oxidation). As Gasteiger and co-workers concluded, the intermediate reacts with ^1^O_2_ again (step III), producing a second carbonyl group and molecule of water. The resulting species then decomposes to produce CO_2_ and CO gas in a 2 : 1 ratio (step IV), with the overall reaction between EC and ^1^O_2_ being EC + 2^1^O_2_ → 2CO_2_ + CO + 2H_2_O.

Previously it has been suggested that EC reacts with ^1^O_2_ to form VC and hydrogen peroxide (H_2_O_2_),^11^ however, neither VC or H_2_O_2_ was found in the EC solution after generating ^1^O_2_. Reaction of VC and ^1^O_2_ in a separate experiment was found to result in the formation of poly VC and several unidentified products, however, none of these products were formed when EC was reacted with ^1^O_2_. Therefore, it is concluded here that the reaction between EC and ^1^O_2_ does not lead to the formation of VC and H_2_O_2_, at least under the conditions studied here and the presence of VC in NMC-based cells at low potentials does not result from a ^1^O_2_-induced reaction.

Since the chemical reactions that generate VC occur at lower potentials than those that involve singlet oxygen, it is likely that these involve Ni^3+^ ions. Singlet oxygen formation will be favoured as the Ni formal oxidation state increases and will likely involve the reduction of Ni^4+^ ions.

#### 4.2.3 Hydrolysis of the electrolyte solution

The formation of HF, fluorophosphate esters (OPF_2_(OR)) and methanol in the cells cycled above the gas evolution onset potential (4.5–4.9 V *vs.* Li^+^/Li for NMC811) indicates that water was generated in these cells, as these species result from the hydrolysis of LiPF_6_ (HF and fluorophosphate esters) and DMC (methanol). The formation of water at these potentials (>80% SOC, >4.4 V for NMC811) further supports an electrolyte decomposition mechanism involving ^1^O_2_, as water forms through the reaction of ^1^O_2_ and EC (see above), and disfavours a mechanism involving a direct electrochemical oxidation step, since these mechanisms do not result in the formation of water.^35–38,92,93^ Furthermore, no proposed soluble products for the electrochemical oxidation of EC could be detected (*e.g.*, acetaldehyde (9.65, 2.13 ppm),^95^ oxirane (ethylene oxide, 2.54 ppm)^96^ and glycolaldehyde (9.68, 6.45 and 4.13 ppm)^97^).

**Scheme 3:**
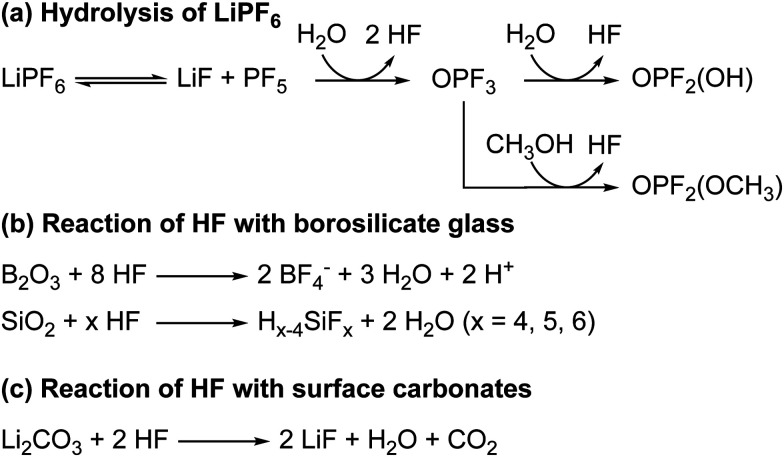
(a) The reaction for the hydrolysis of LiPF_6_ to form HF and fluorophosphate esters (OPF_2_(OR)). (b) The reactions of HF with the borosilicate glass fibre separator to form boron and silicon fluorides. (c) The acid–base reaction of HF with lithium carbonate to form lithium fluoride, H_2_O and CO_2_.

The hydrolysis of the LiPF_6_ results in the formation of fluorophosphate esters and HF in the electrolyte solution. HF can then attack the borosilicate glass fibre separator, producing boron and silicon fluoride species, as is observed by the presence of BF_4_^−^ and SiF_*x*_ in the electrolyte, as reported previously. The formation of these species has been reported before and are summarised in Scheme 3.^98^ It is noted that borosilicate glass fibre separators are not used in commercial cells. In previous work on LiCoO_2_ electrodes by some of the authors,^63^ we reported the formation of glycolic acid at potentials above the onset of gas evolution; however, this assignment is found to be incorrect as the chemical shift for glycolic acid in DMSO (measured in a separate experiment) was determined to be 3.92 ppm (Fig. S30, ESI†). Instead, the signal at 3.99 ppm is now assigned to the fluorophosphates ester OPF_2_(OCH_3_), based on the appearance of a signal at 3.98 ppm in the ^1^H NMR spectra of LP30 with 2 vol% methanol added (Fig. 10).

**Scheme 4:**
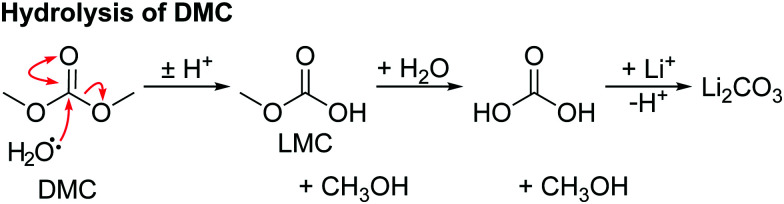
The reaction for the hydrolysis of dimethyl carbonate (DMC) to form methanol and lithium carbonate. Lithium methyl carbonate (LMC) and dihydrogen carbonate are intermediates.

The hydrolysis of DMC results in the formation of methanol and lithium carbonate as shown in Scheme 4 and reported in our previous work.^63^ No evidence for the hydrolysis of EC is observed, as neither lithium ethylene monocarbonate (LEMC; 4.08 and 3.55 ppm) ethylene glycol (EG; 3.44 and 3.25 ppm) could be detected in the ^1^H NMR spectra (Fig. 4). This is ascribed to EC being less susceptible to hydrolysis, in part because its hydrolysis product, LEMC, can undergo a ring-closing reaction, reforming the EC molecule.

#### 4.2.4 Oxidation of methanol

The presence of formaldehyde and formic acid in cells cycled to very high potentials (4.7–4.9 V *vs.* Li^+^/Li for NMC811) is ascribed to the oxidation of methanol at the transition metal oxide surface. This is supported by the formation of these two species in NMC811/LFP cells cycled with an electrolyte solution containing 2 vol% methanol: formaldehyde was detected in cells where the NMC electrode was cycled to potentials as low as 4.1 V (*vs.* Li^+^/Li), and formic acid was observed when the NMC is cycled to 4.7 V (Fig. 10). The degree of oxidation of methanol may be related to the oxide phase at the surface of the transition metal oxide (layered, spinel or rock salt) and the availability of surface oxygen ions.^99^

The absence of formaldehyde in NMC811/LFP cells cycled up to 4.5 V *vs.* Li^+^/Li, suggest that no significant amount methanol was produced in those cells. As discussed above, methanol is formed through the hydrolysis of DMC with water generated from EC oxidation. The absence of methanol at this potential may seem surprising, as gas evolution (which indicates EC oxidation by ^1^O_2_ and thus the formation of water) already occurs at 4.4 V *vs.* Li^+^/Li for NMC811. The cell where the NMC electrode is cycled to 4.5 V does, however, contain the hydrolysis products of LiPF_6_ (HF and fluorophosphate esters), suggesting that water is formed at this potential. The absence of methanol is thus ascribed to the faster hydrolysis of LiPF_6_*versus* DMC and/or the small amount of water formed at this potential.

**Scheme 5:**
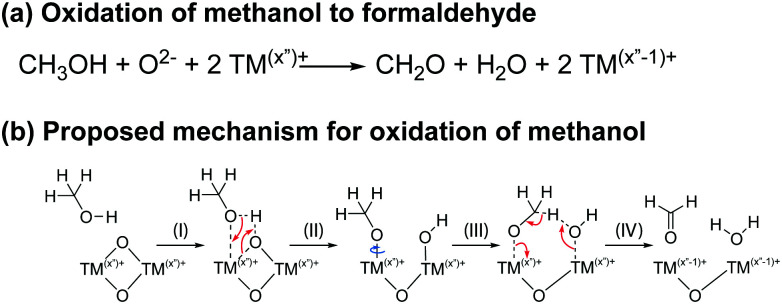
The proposed reaction (top) and mechanism (bottom) for the oxidation of methanol to formaldehyde at the surface of the NMC electrode. The oxidation states of the transition metal ions in this scheme are illustrative and other oxidation states are possible, the NMCs comprising a mixture of Ni^3+^ and Ni^4+^ (in the bulk) at the potentials at which these reactions occur.

The proposed reaction and mechanism for the chemical oxidation of methanol to formaldehyde are given in Scheme 5. Methanol is oxidised by transition metal ions on the surface of the electrode, resulting in the formation of formaldehyde and water, and the reduction of the transition metal ion. The proposed mechanism is loosely based on those suggested for the oxidation of methanol on iron molybdate and ruthenium complex catalysts:^100,101^ an acid–base reaction between methanol and a bridging oxygen results an methoxy group adsorbed to the TM ion and terminal OH group (step I and II). Nucleophilic attack by the OH oxygen on the methoxy group results in formation of formaldehyde and water, and the reduction of the transition metal ion at the surface (step III and IV).

**Scheme 6:**
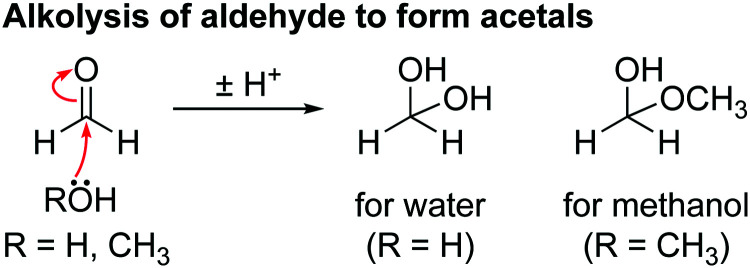
The reaction for the hydration of aldehydes by water (R = H) or methanol (R = CH_3_) to form acetals.

The presence of acetal species (methanediol and methoxymethanol) is ascribed to the reaction of water and methanol with formaldehyde. Scheme 6 shows the proposed mechanism for the hydration of formaldehyde: nucleophilic attack by water or methanol on the carbonyl carbon of formaldehyde result in the formation of methanediol and methoxymethanol, respectively.

#### 4.2.5 Formation of FEC

**Scheme 7:**
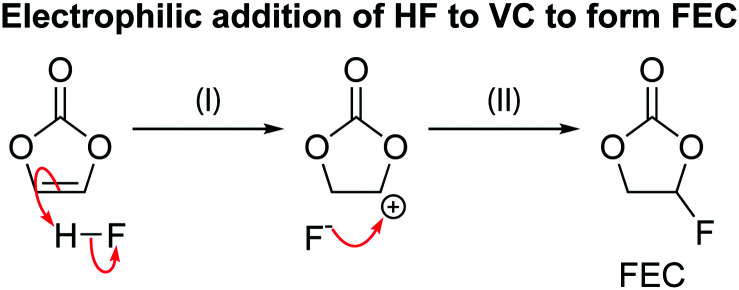
The reaction for electrophilic addition of hydrofluoric acid (HF) to vinylene carbonate (VC) to form fluoroethylene carbonate (FEC).

The formation of FEC in cells cycled to high potentials is tentatively ascribed to reaction between VC and HF (Scheme 7), a proposal that is supported by the observation of FEC at the same potential at which HF is produced. The first step involves electrophilic addition of the proton to the electron-rich double bond of VC to form a carbocation intermediate. The second step involves nucleophilic attack of the fluoride ion on the carbocation to form FEC.

### 4.3 Summary of reactions at the positive electrode

An overview of the two routes for electrolyte decomposition at the NMC electrode was given in Fig. 11 and is now revisited in the context of the reaction schemes proposed above. At low potentials (*i.e.*, <80% SOC), the carbonate solvent, EC, is dehydrogenated to VC, reducing the transition metal ions at the surface and producing protons which convert the NMC oxide surface to an oxy-hydroxide (Scheme 1). At high potentials (*i.e.*, >80% SOC), ^1^O_2_ released from the transition metal oxide lattice reacts with EC to produce CO_2_, CO and water (Scheme 2). This water can then hydrolyse the electrolyte, resulting in the formation of fluorophosphates and HF (Scheme 3a) and the HF can react further with the glass fibre separator to produce boron and silicon fluorides (Scheme 3b) and with VC to form FEC (Scheme 7). The water (from EC + ^1^O_2_) also slowly hydrolyses DMC to form methanol (Scheme 4), which can subsequently be oxidised to formaldehyde and formic acid (Scheme 5). Both water and methanol can hydrate the formaldehyde to produce acetals (Scheme 6). All of the decomposition reactions at high potentials (>80% SOC) are initiated by the formation of water from the reaction between ^1^O_2_ and EC. Although all these reactions are expected to happen regardless of the negative electrode material, some of the reaction products can diffuse away and react with the negative electrode, effect known as ‘electrodes’ cross-talk’, as discussed below.

### 4.4 Further reactions of the electrolyte decomposition products at the negative electrode

The comparison of the decomposition products formed in NMC/LFP cells with those formed in NMC/graphite cells reveals that fewer decomposition products are present in the NMC/graphite “full cells”. The only signals detected in the electrolyte from the NMC811/graphite cells were those of lithium formate and formic acid, in addition to the signals for LEDC and methanol resulting from reactions at the graphite electrode. The signals from formaldehyde, VC, FEC, acetals, HF, BF_*x*_ and SiF_*x*_, fluorophosphates esters could not be detected. Except for that of lithium formate, no new signals were observed. This implies that there were no reactions between the graphite electrode or SEI and the decomposition products from the NMC electrode that resulted in (partial) dissolution of SEI components or modification of the decomposition products (*i.e.*, no soluble reaction products were formed).

The presence of lithium formate and formic acid is ascribed to the reduction of CO_2_ at the graphite electrode,^102,103^ rather than the oxidation of methanol at the NMC electrode, as the intermediate for methanol oxidation (formaldehyde) was not detected. The CO_2_ is produced through the chemical oxidation of EC by ^1^O_2_ and Li_2_CO_3_ decomposition at the NMC electrode, and, subsequently, CO_2_ diffuses to the graphite electrode, where it is consumed.^33,104^ Even though methanol could arise from the hydrolysis of DMC (induced at the positive electrode), the quantities observed in the “full cell” are similar to those observed in the LFP/graphite cell and is thus attributed to the reduction of DMC at the graphite electrode.

The absence of the decomposition products formed at the NMC electrode in the NMC/graphite “full cells” is ascribed to the consumption of water, formed through the reaction between ^1^O_2_ and EC (which initiates the electrolyte decomposition chain), at the graphite electrode. Two alternative reaction pathways for water involving the graphite electrode are the hydrolysis of SEI components and the reduction of water to LiOH and H_2_. As no hydrolysis products of SEI components are detected in the electrolyte solution, it is concluded that water is reduced to LiOH and H_2_ at the graphite surface, thickening the SEI with inorganic components.^105–107^ Trace quantities of HF present in the pristine electrolyte solution will be reduced in a similar way to H_2_ and LiF.^108^ Reactions of HF with inorganic species such as Li_2_CO_3_ to form CO_2_, HF and H_2_O (and LiOH+ H_2_) are also possible. The absence of VC (the only product not formed as a result of the reaction between ^1^O_2_ and EC), is ascribed to the reduction of VC at the graphite electrode, a reaction which is known to occur at 1.1 V *vs.* Li^+^/Li.^79,109^ These results also suggest that the SEI on graphite in this cell is not completely passivating, as species in the electrolyte solution can still be reduced. These side reactions that occur at the graphite negative electrode, with compounds formed at the NMC electrode, have severe consequences in the lifetime of the battery, as discussed below.

### 4.5 Implications of electrolyte decomposition routes and products

Although it is desirable to cycle the batteries to high cell voltages, the results above show that electrolyte oxidation becomes more significant as the potential of the NMC electrode is increased, and as the time spent at this potential is increased.

The formation of VC at low cell voltages is suggested to be less detrimental to the cell's lifetime: VC has been shown to be an effective electrolyte additive to produce a stable SEI on graphite electrodes, but when used at high concentrations (>2%) it causes an impedance rise at the negative electrode.^109^ A rough quantification (from NMR integration) revealed that ∼100 ppm VC is present in NMC/LFP cells after 10 cycles, making it an unlikely cause for the capacity fade. Furthermore, the potential at which VC is formed (<4.1 V) is lower than when the increased capacity fade is observed. The formation of VC could be prevented by introducing a coating on the NMC surface that would prevent direct contact between the electrolyte solution and the NMC particles.^45^ The formation of PO_2_F_2_^−^ is also thought to be less harmful to the cell, as LiPO_2_F_2_ is routinely used as an additive in these systems,^110,111^ and thus some of the decomposition products may actually be beneficial.

The decomposition products formed at the positive electrode at high potentials (>80% SOC) appear to be consumed (*i.e.*, reduced and/or deposited) on the surface of the graphite electrode, as no (new) soluble products were detected. The deposition of products on graphite surface further increases the cell impedance, and over many charge–discharge cycles, this may limit the ion transport to the bulk of the negative electrode, making a significant contribution to the capacity fade of the cell.^21,24,26,28^ The small amount of decomposition products formed at ≥80% SOC (total products ∼1000 ppm, quantified by NMR integration, after 10 cycles between 4.7–3.0 V) may not explain all of the capacity loss that is generally observed. Even though no dissolution of the SEI was detected, it is still possible that the decomposition products modified the SEI (without dissolving it) exposing a fresh surface for continued electrolyte reduction. For example, acid–base reactions between the acidic species formed at the NMC electrode and the basic components of the SEI could increase the porosity of the SEI (*e.g.*, HF will react readily with Li_2_CO_3_), allowing for the electrolyte solution to penetrate into the SEI and be reduced at the graphite surface. This could lead to a much greater decrease in the available lithium inventory than the consumption of the decomposition products alone. A more in-depth study of the reaction between the decomposition products formed at the positive electrode and the SEI on the graphite electrode could clarify this.

We note that one major source of electrolyte decomposition is water and thus, the formation of these decomposition products could be mitigated by introducing a water-scavenging electrolyte additive or separator. Another approach would be the use of water-stable salts (unlike LiPF_6_, *e.g.*, lithium borates: LiBF_4_ or LiBOB; lithium imides: LiTFSI, LiFSI) or electrolytes resistant to attack by singlet oxygen (unlike EC). Alternatively, a coating or graded particle that hinders or even prevents the release of oxygen from the lattice and transition metal dissolution, could be used. While DMC does appear to be stable against singlet oxygen, future studies will investigate the stability of other linear carbonates and PC.

## 5 Conclusion

This work focused on understanding the electrolyte decomposition reactions occurring at NMC electrodes and the further reaction of decomposition products at the negative electrode (graphite). Using solution NMR spectroscopy, the soluble decomposition products formed at NMC electrodes and in NMC/graphite cells were identified for the first time. The NMR results were complemented by gas analysis experiments to yield a more complete picture of the electrode/electrolyte decomposition reactions. To explore various possible decomposition mechanisms, the electrolyte solvent was reacted with isotopically labelled singlet oxygen (^17^O-labelled ^1^O_2_, produced from ^17^O-labelled ^3^O_2_*via* a photosensitiser).

A comparison of the electrolyte decomposition products formed at NMC electrodes at high SOC with those formed through the reaction with ^1^O_2_ showed that the detected products formed at high potentials in NMC-based cells are consistent with ^1^O_2_ being released from the lattice and reacting with the electrolyte solution.

The identified decomposition products revealed two distinct routes for electrolyte decomposition, each with a different onset potential. At low potentials (*i.e.*, <80% SOC), EC is dehydrogenated to VC, which may be coupled to the reduction of transition metal ions at the surface, but without the release of any gaseous decomposition products. The second, and more destructive, mechanism occurs when the material reaches 80% SOC, and ^1^O_2_ is released from the lattice: ^1^O_2_ chemically oxidises the electrolyte solvent (EC) to produce H_2_O, CO_2_ and CO. The formed water then hydrolyses the electrolyte solution and initiates a series of reactions that were so far unknown to occur in lithium-ion batteries: the oxidation of alcohols to their corresponding aldehydes and carboxylic acids, the hydration of aldehydes to form acetals, and the formation of FEC from VC – these products being identified by NMR. The NMC/graphite cells revealed fewer decomposition products, which is attributed to the reduction of water to form LiOH and H_2_ at the graphite (SEI) surface. The increased parasitic reactions at the negative electrode decrease the available lithium inventory and may contribute to the observed capacity fade when the cell is cycled to higher voltages. However, the interaction between the decomposition products and the SEI may be more complex (involving modifications of the SEI and the exposure of fresh surface leading to continual electrolyte reduction) and will be the subject of future studies.

The release of ^1^O_2_ drives the electrolyte decomposition reactions at the NMC electrode, a process which is intrinsically linked to reaching high SOC and thus higher cell capacities. Understanding the mechanism *via* which oxygen is released and developing strategies to prevent this process will be valuable for improving the lifetime of lithium-ion batteries, particularly those containing the more environmentally-sustainable Ni-rich positive electrode materials.

## Author contributions

Bernardine L. D. Rinkel: conceptualisation; methodology; investigation; writing – original draft; writing – review & editing. J. Padmanabhan Vivek: methodology; investigation; writing – review & editing. Nuria Garcia-Araez: funding acquisition; supervision; writing – review & editing. Clare P. Grey: conceptualisation; funding acquisition; supervision; writing – review & editing.

## Conflicts of interest

There are no conflicts to declare.

## Supplementary Material

EE-015-D1EE04053G-s001
