# Supplementary material for: Two electrolyte decomposition pathways at nickel-rich cathode surfaces in lithium-ion batteries
Source: Energy Environ Sci. 2022 Jul 5;15(8):3416–38. doi: 10.1039/d1ee04053g (PMC9368649; doi:10.1039/d1ee04053g)
Supplement: EE-015-D1EE04053G-s001 [file EE-015-D1EE04053G-s001.pdf]

Electronic Supplementary Material (ESI) for Energy & Environmental Science.
This journal is © The Royal Society of Chemistry 2022

Two electrolyte decomposition pathways at nickel-rich cathode surfaces in lithium-ion batteries

Supplementary information

Bernardine L. D. Rinkel,[1] J. Padmanabhan Vivek,[2,3] Nuria Garcia-Araez,[2,3] Clare P. Grey[1,3]

[1]*Department of Chemistry, University of Cambridge, Cambridge CB2 1EW, United Kingdom*
[2]*Department of Chemistry, University of Southampton, Southampton SO17 1BJ, United Kingdom*
[3]*The Faraday Institution, Harwell Campus, Didcot OX11 0RA, United Kingdom*

1. Comparison of potential profiles for NMC811/Li and NMC811/Li_xFePO_4 cells.............2
2. Setup for generating singlet oxygen ($^1O_2$) 3
3. Additional operando pressure measurements............4
4. Additional on-line electrochemical mass spectrometry (OEMS) measurements 5
5. Three electrode measurements............7
6. Additional ^{1}H, ^{19}F and ^{17}O NMR spectra............8
   6.1. DMSO-*d*$_6$............8
   6.2. NMC811/delithiated LFP cells............9
   Comparison of glass fibre with polypropylene............11
   6.3. NMC622/delithiated LFP cells............13
   6.4. NMC532/delithiated LFP cells............15
   6.5. NMC111/delithiated LFP cells............17
   6.6. LCO/delithiated LFP cells............19
   6.7. NMC/graphite and LCO/graphite cells............21
   6.8. Reactions between singlet oxygen and the carbonate solvent............23
   6.9. Assignment of the ^{1}H NMR signal at 3.98 ppm............26
7. References............27

1. Comparison of potential profiles for NMC811/Li and NMC811/Li_xFePO_4 cells

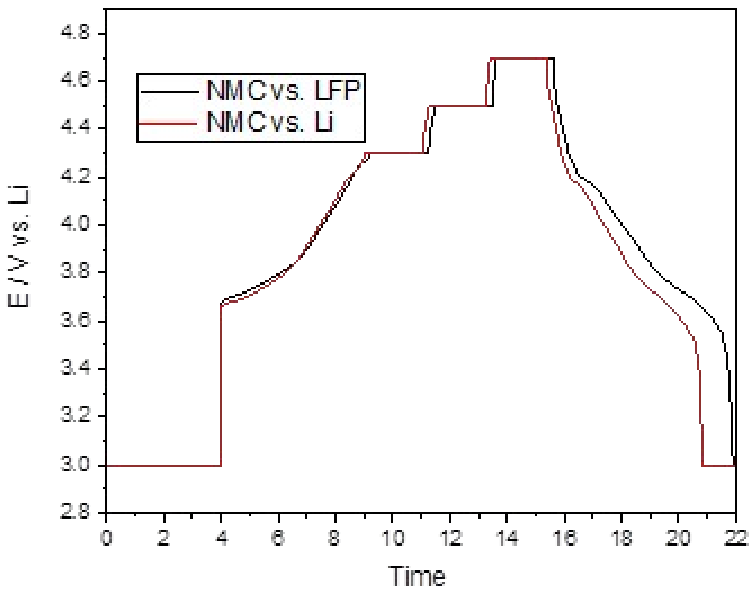

Figure S1. Comparison of the potential profiles of NMC811 electrodes in Li half-cells (data in Figure S6) and in cells with a $Li_{0.25}FePO_4$ counter electrode (data in figure S5) using a conversion of potential that takes into account the potential of the $Li_{0.25}FePO_4$ electrode (3.45 V vs. Li^+/Li).

2. Setup for generating singlet oxygen ($^1O_2$)

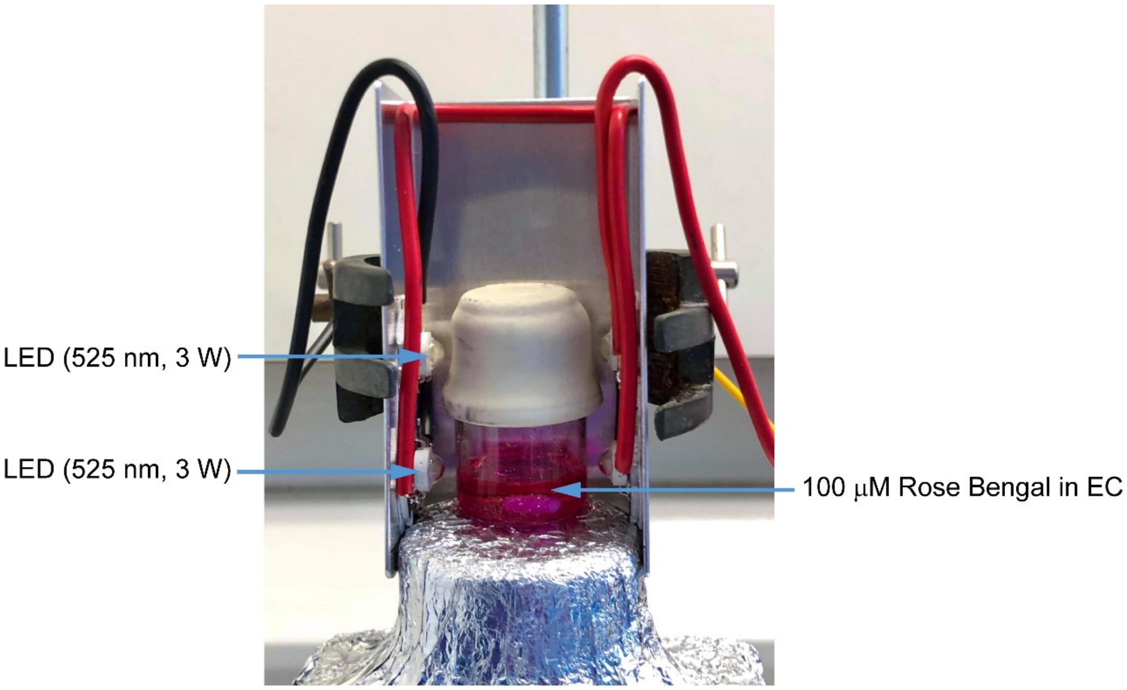

Figure S2. The setup used to generate singlet oxygen.

3. Additional operando pressure measurements

To deconvolute the contribution of the EC and DMC solvents to the pressure increase observed in NMC811/Li cells made with an LP30 electrolyte solution, the measurements were repeated using a 'DMC-only' or an 'EC-only' electrolyte solution. A higher salt concentration (1.5 M vs 1 M) was used for the EC-only electrolyte to ensure that the solution was liquid at room temperature.

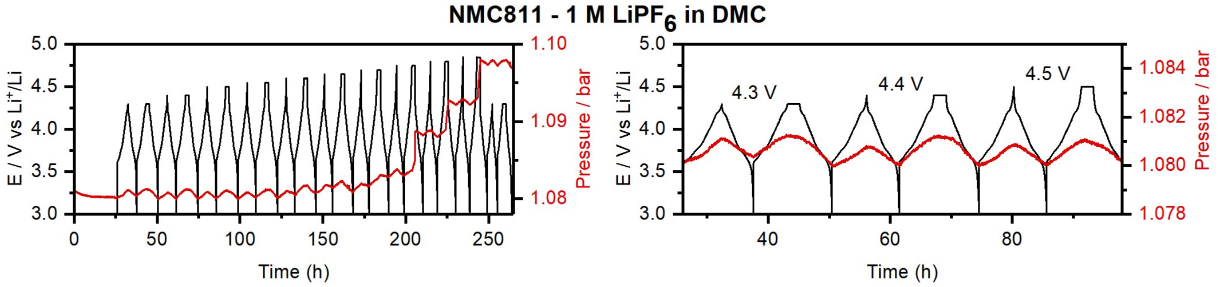

Figure S3. Operando pressure data for an NMC811/Li cells using a 1 M $LiPF_6$ in DMC electrolyte. The cell was cycled between 3.0 V and a series of increasing upper cut-off potentials (4.3–4.85 V), after which it were cycled to 4.3 V again; where the potential was stepped by 0.1 V every two cycles until 4.5 V, whereafter the step-size was reduced to 0.05 V. For every cut-off potential value, the second cycle included a 2-hour potential hold at the top of charge. The internal cell pressure and potential-time data are shown in red and black, respectively. An expanded view of the data is shown on the right. The active material mass loading was 3 mg cm^{-2}.

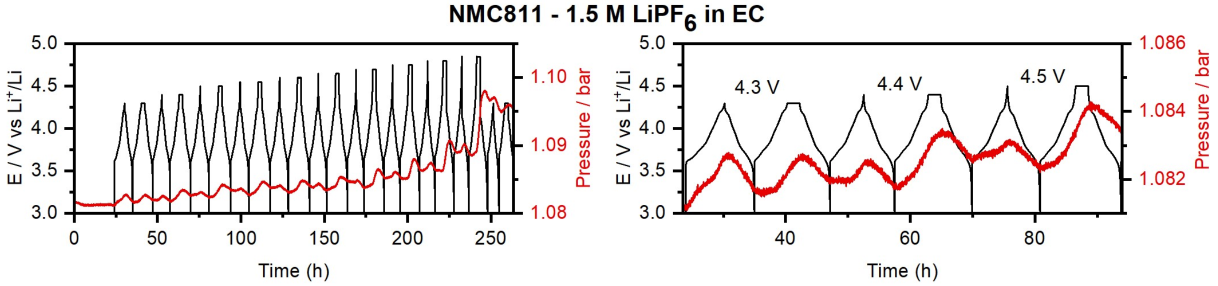

Figure S4. Operando pressure data for an NMC811/Li cells using a 1.5 M $LiPF_6$ in EC electrolyte. The cell was cycled between 3.0 V and a series of increasing upper cut-off potentials (4.3–4.85 V), after which it were cycled to 4.3 V again; where the potential was stepped by 0.1 V every two cycles until 4.5 V, whereafter the step-size was reduced to 0.05 V. For every cut-off potential value, the second cycle included a 2-hour potential hold at the top of charge. The internal cell pressure and potential-time data are shown in red and black, respectively. An expanded view of the data is shown on the right. The active material mass loading was 3 mg cm^{-2}.

4. Additional on-line electrochemical mass spectrometry (OEMS) measurements

To confirm that the evolution of C_2H_4 and CO observed at low cell potentials in NMC811/Li cells originates from electrolyte reduction reactions at the lithium metal electrode, the OEMS measurements were repeated using a partially delithiated Li_xFePO_4 (LFP) electrode.

The amount of oxygen produced in the NMC811/LFP cell is twice as high as that produced in the NMC811/Li cell (100 vs 50 ppm). We attribute this difference to the consumption of the generated oxygen by the lithium metal electrode to form lithium oxides.

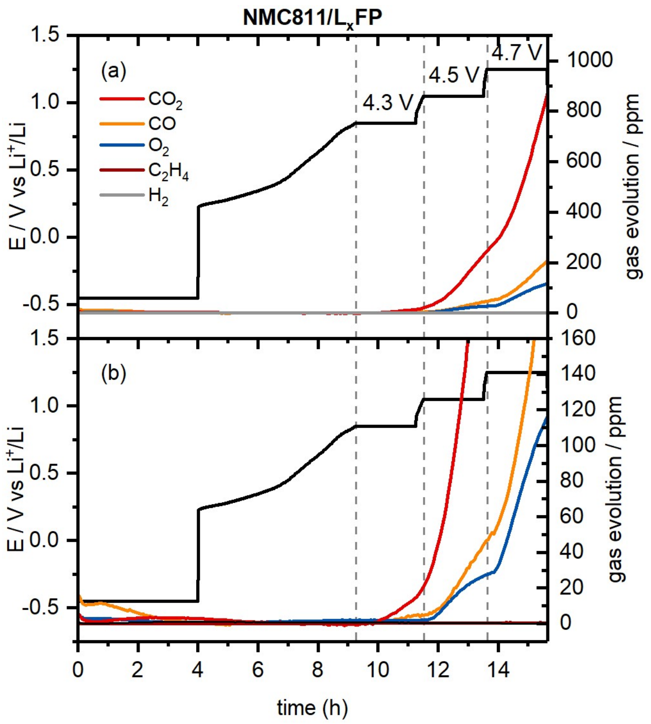

Figure S5. (a) Gas evolution in a NMC811/delithiated-LFP cell charged from 3.0 to 4.7 V (vs Li^+/Li) with a 2-hour potential hold at 4.3, 4.5 and 4.7 V as measured by online-electrochemical mass spectrometry (OEMS). The cell potential-time data is given in black. The gas concentration in the cell head space are given in ppm for CO_2 (m/z = 44), CO (m/z = 28), O_2 (m/z = 32), C_2H_4 (m/z = 26) and H_2 (m/z = 2). (b) Enlarged view of the gases evolved at low concentrations (between 0–700 ppm). The active material mass loading was 3 mg cm^{-2} for the NMC electrode and 15 mg cm^{-2} for the delithiated LFP counter-electrode.

The formation of CO_2 or H_2 at low potentials, < 4.3V vs. Li^+/Li, is negligible in the NMC811/Li cells (Figure 3 in the main text and Figure S6). Previous work has shown that the reduction of water traces at the anode produces H_2 and hydroxide ions, which then promote the decomposition of the organic electrolyte forming CO_2.[10,63,67] These processes are absent in the NMC/LFP cell in Figure S5, as expected, because the potential of the LFP counter electrode is too high to induce water reduction. However, these processes are also

absent in the NMC/Li cell in figure 3, thus demonstrating the thorough drying of the electrolyte and all cell components in our experiments."

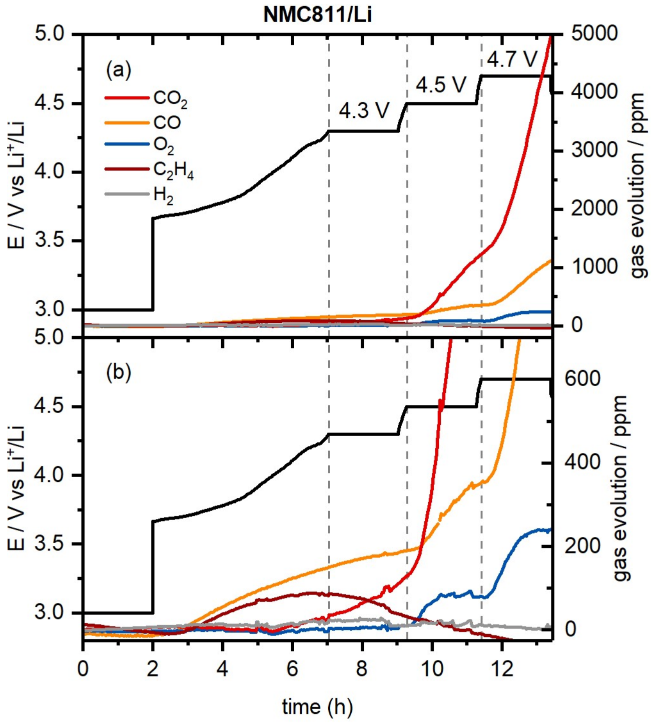

Figure S6. (a) Gas evolution in a NMC811/Li cell charged from 3.0 to 4.7 V (vs Li^+/Li) with a 2-hour potential hold at 4.3, 4.5 and 4.7 V as measured by online-electrochemical mass spectrometry (OEMS). The cell potential-time data is given in black. The gas concentration in the cell head space are given in ppm for CO_2 (m/z = 44), CO (m/z = 28), O_2 (m/z = 32), C_2H_4 (m/z = 26) and H_2 (m/z = 2). (b) Enlarged view of the gases evolved at low concentrations (between 0–700 ppm). The active material mass loading was 9 mg cm^{-2}.

5. Three electrode measurements

The potential the NMC electrode reaches in NMC/delithiated-LFP cells was determined using a three-electrode cell, where lithium metal was used as the reference electrode.

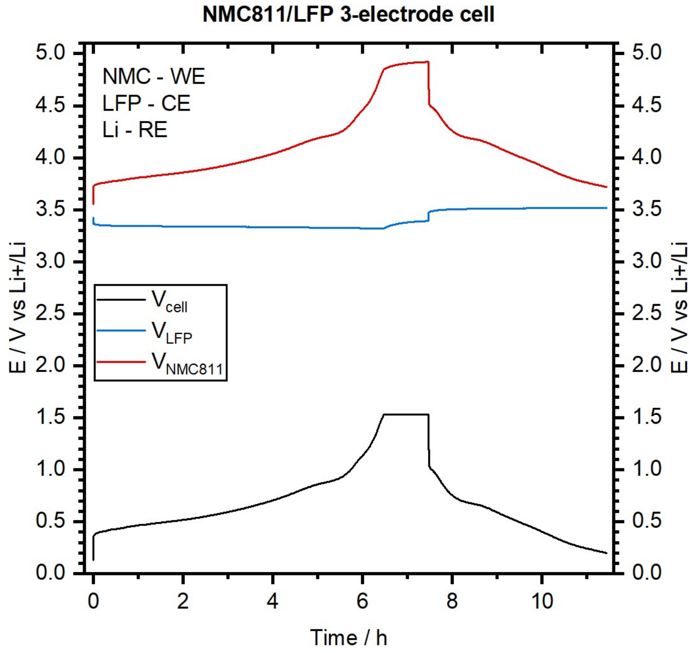

Figure S7. Galvanostatic charge and discharge curve for a three-electrode NMC811/Li/delithiated LFP cell, cycled at a C/5 rate between 0.2–1.53 V_{cell}, which was found to correspond to a potential window of 3.7–4.9 V vs Li/Li^+ for the NMC811 electrode.

6. Additional ^{1}H, ^{19}F and ^{17}O NMR spectra

6.1. DMSO-d_6

To help distinguish between ^{1}H NMR signals arising from electrolyte decomposition products and those originating from impurities, ^{1}H NMR spectra of each batch of DMSO-d_6 were acquired. The DMSO-d_6 used in this work was 99% chemically pure. Individually sealed ampoules of DMSO-d_6 (0.75 mL) were used to avoid any absorption of moisture or further deterioration of the solvent.

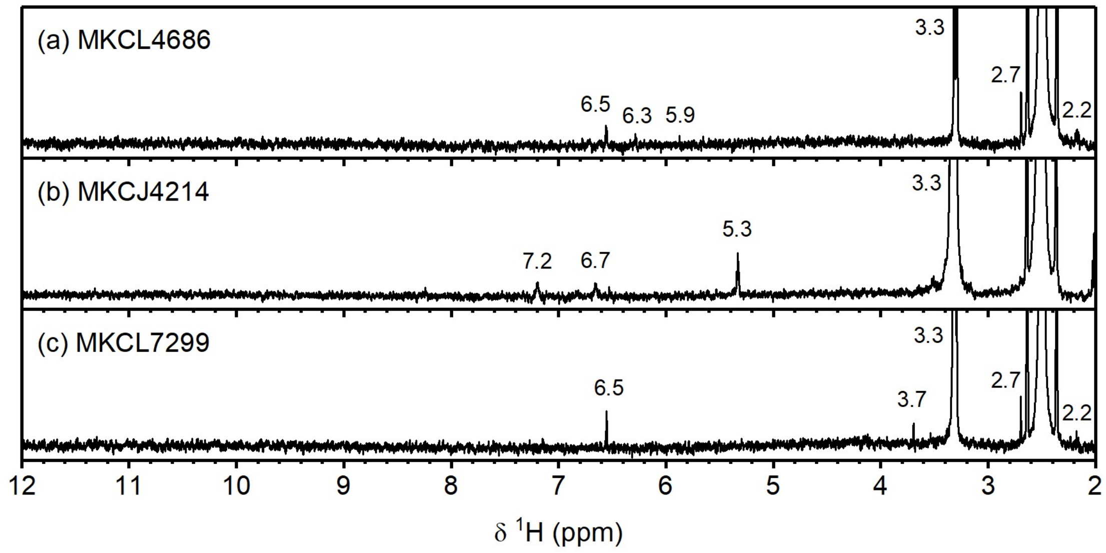

Figure S8. ^{1}H NMR spectra of three batches of DMSO-d_6 used in this work.

6.2. NMC811/delithiated LFP cells

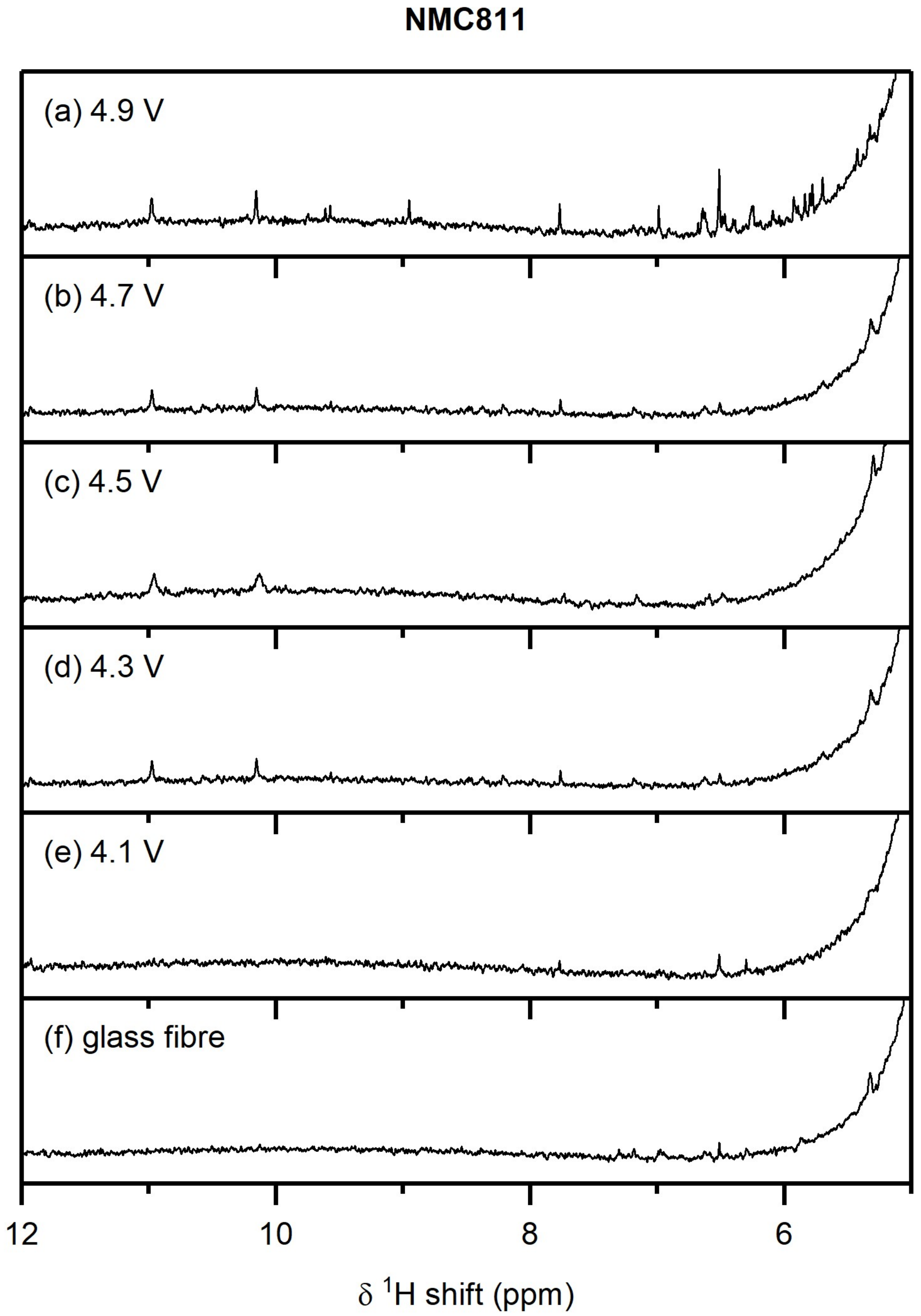

Figure S9. ^{1}H NMR spectra of electrolyte solutions extracted from NMC811/LFP cells after 10 cycles, where the cell cut-off voltages were chosen so the NMC electrode was cycled between (a) 4.9 V, (b) 4.7 V, (c) 4.5 V, (d) 4.3 V, (e) 4.1 V and 3.0 V vs Li^+/Li, and (f) pristine electrolyte solution. The cells were cycled at rate of C/5 in constant current-constant voltage (CCCV) mode.

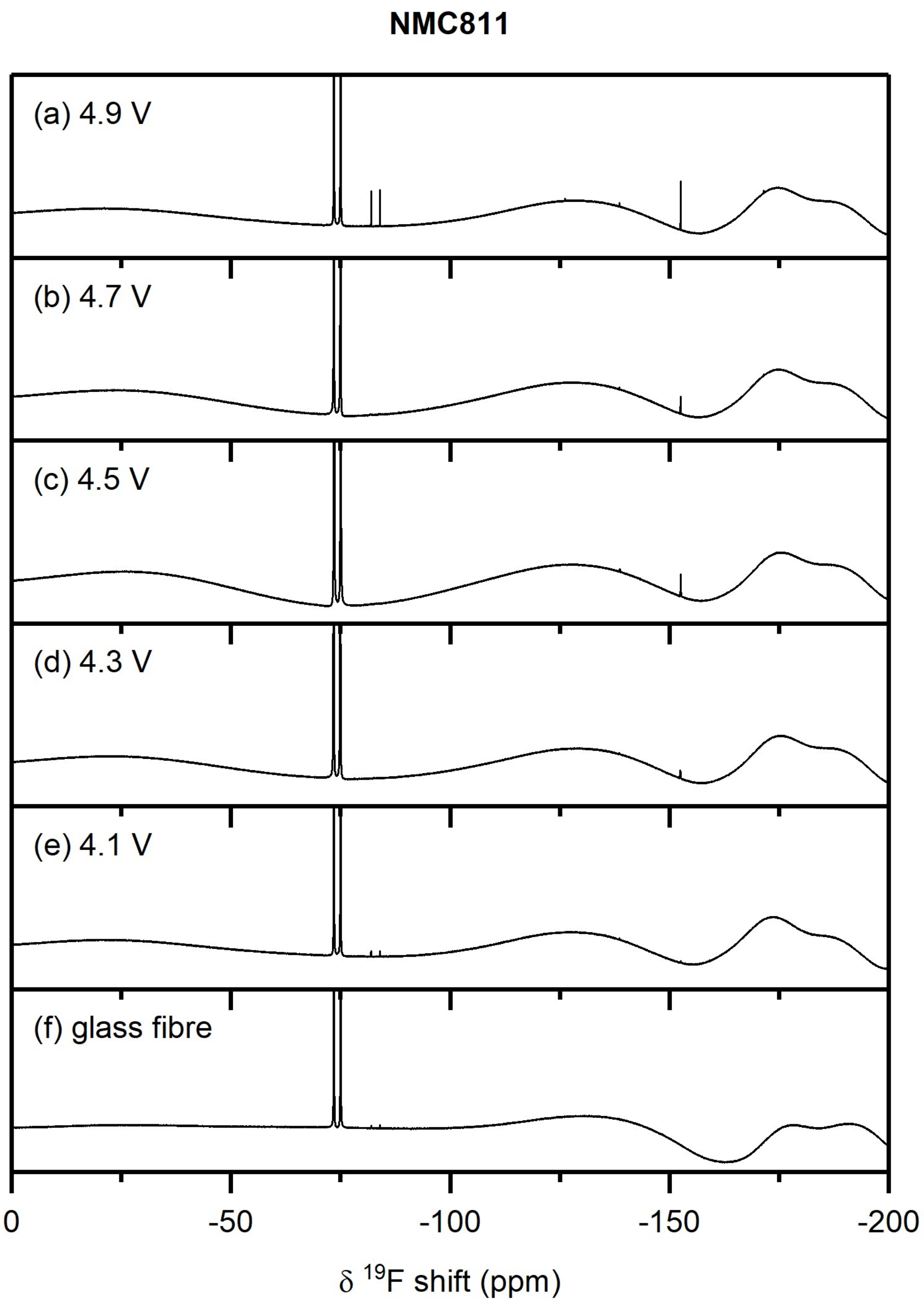

Figure S10. ^{19}F NMR spectra of electrolyte solutions extracted from NMC811/LFP cells after 10 cycles, where the cell cut-off voltages were chosen so the NMC electrode was cycled between (a) 4.9 V, (b) 4.7 V, (c) 4.5 V, (d) 4.3 V, (e) 4.1 V and 3.0 V vs Li^+/Li, and (f) pristine electrolyte solution. The cells were cycled at rate of C/5 in constant current-constant voltage (CCCV) mode.

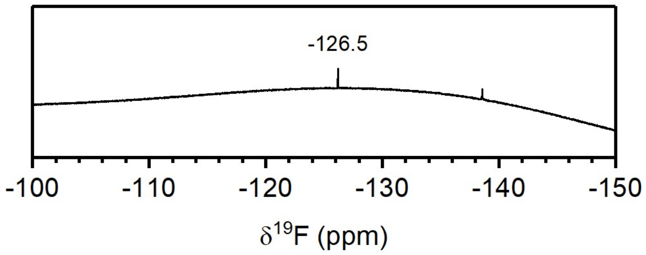

Figure S11. Expanded region of the ^{19}F NMR spectra shown in Figure 4.6:electrolyte solution extracted from an NMC811/LFP cell after 10 cycles, where the cell cut-off voltages were chosen so the NMC electrode was cycled between (a) 4.9 V and 3.0 V vs Li^+/Li. The cell was cycled at rate of C/5 in constant current-constant voltage (CCCV) mode.

Comparison of glass fibre with polypropylene

The cell made with a polypropylene (PP) separator (Celgard 3501; b) shows the presence of presence of methanol, formic acid, acetals and $OPF_2(OCH_3)$. This indicates that DMC is hydrolysed, and thus demonstrates that water is also formed in cells without a glass fibre (G/F) separator (and so the water cannot originate from reactions of HF with the separator, but instead must be formed in a different way). We therefore attribute the formation of water to the chemical oxidation of EC by singlet oxygen.

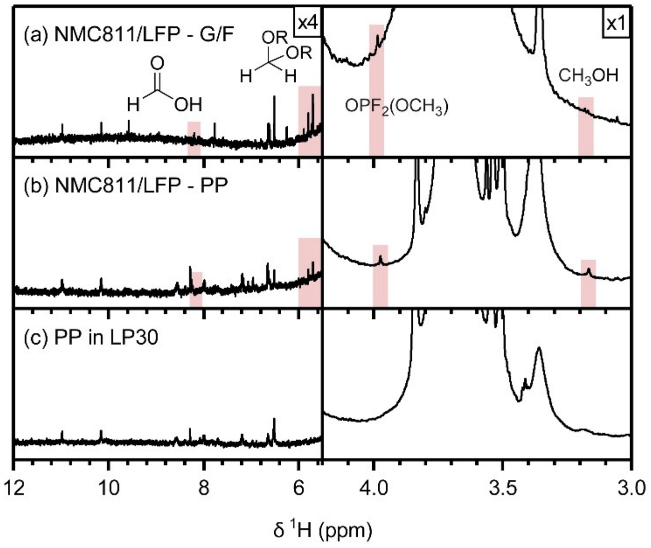

Figure S12. ^1H NMR spectra of electrolyte solutions extracted from NMC811/Li_xFePO_4 cells after 10 cycles, where the cell cut-off voltages were chosen so the NMC electrode was cycled between (a) 4.9 V and 3.0 V vs Li^+/Li, assembled with a (a) glass fibre (G/F) and (b) polypropylene (PP) separator (Celgard 3501). (c) ^1H NMR spectrum of a Celgard separator soaked in LP30 solution in a coin cell casing (same components as NMC811/LFP, but without the electrodes) for the same duration.

No ^{19}F NMR signal is seen for SiF_x and only a small signal is seen for BF_4^- when a PP separator is used instead of a G/F one. The small amount of BF_4^- most likely comes from the

glass NMR tube or the glass vial the separator is placed in to extract the electrolyte solution with DMSO. The ^{19}F NMR spectra show that the SiF_x and BF_4^- species observed in cells assembled with a G/F separator originate from the glass fibre, and the ^{19}F NMR signal intensity of SiF_x/BF_4^- can be used as a measure for the amount of HF formed in the cell.

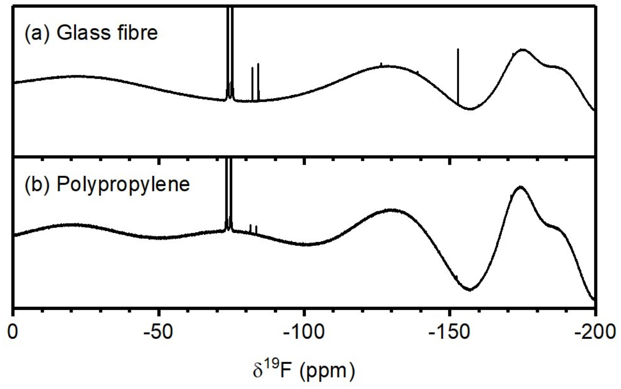

Figure S13. ^{19}F NMR spectra of electrolyte solutions extracted from NMC811/Li_xFePO_4 cells after 10 cycles, where the cell cut-off voltages were chosen so the NMC electrode was cycled between (a) 4.9 V and 3.0 V vs Li^+/Li, assembled with a (a) glass fibre and (b) polypropylene separator (Celgard 3501).

6.3. NMC622/delithiated LFP cells

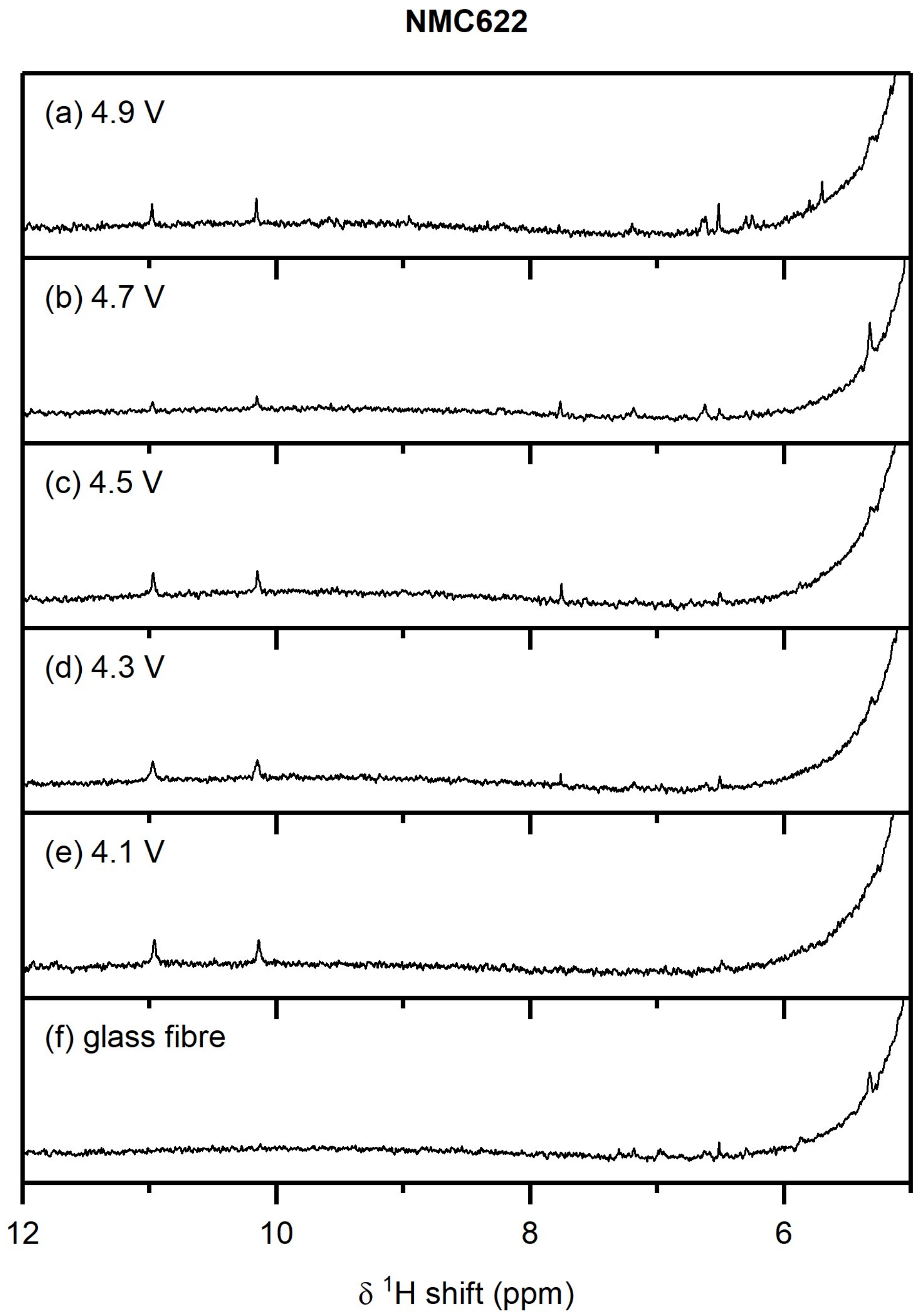

Figure S14. ^{1}H NMR spectra of electrolyte solutions extracted from NMC622/LFP cells after 10 cycles, where the cell cut-off voltages were chosen so the NMC electrode was cycled between (a) 4.9 V, (b) 4.7 V, (c) 4.5 V, (d) 4.3 V, (e) 4.1 V and 3.0 V vs Li^+/Li, and (f) pristine electrolyte solution. The cells were cycled at rate of C/5 in constant current-constant voltage (CCCV) mode.

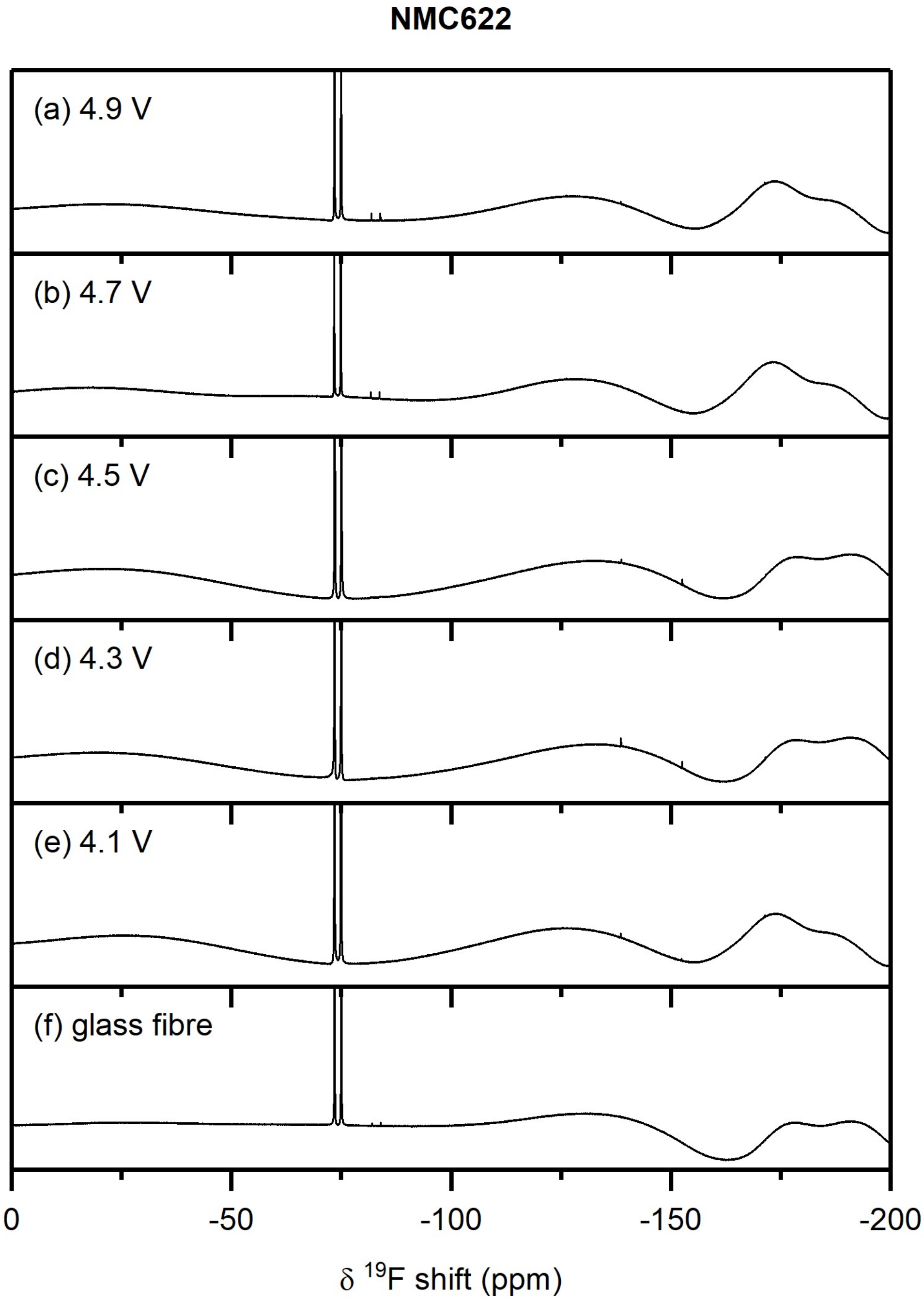

Figure S15. ^{19}F NMR spectra of electrolyte solutions extracted from NMC622/LFP cells after 10 cycles, where the cell cut-off voltages were chosen so the NMC electrode was cycled between (a) 4.9 V, (b) 4.7 V, (c) 4.5 V, (d) 4.3 V, (e) 4.1 V and 3.0 V vs Li^+/Li, and (f) pristine electrolyte solution. The cells were cycled at rate of C/5 in constant current-constant voltage (CCCV) mode.

6.4. NMC532/delithiated LFP cells

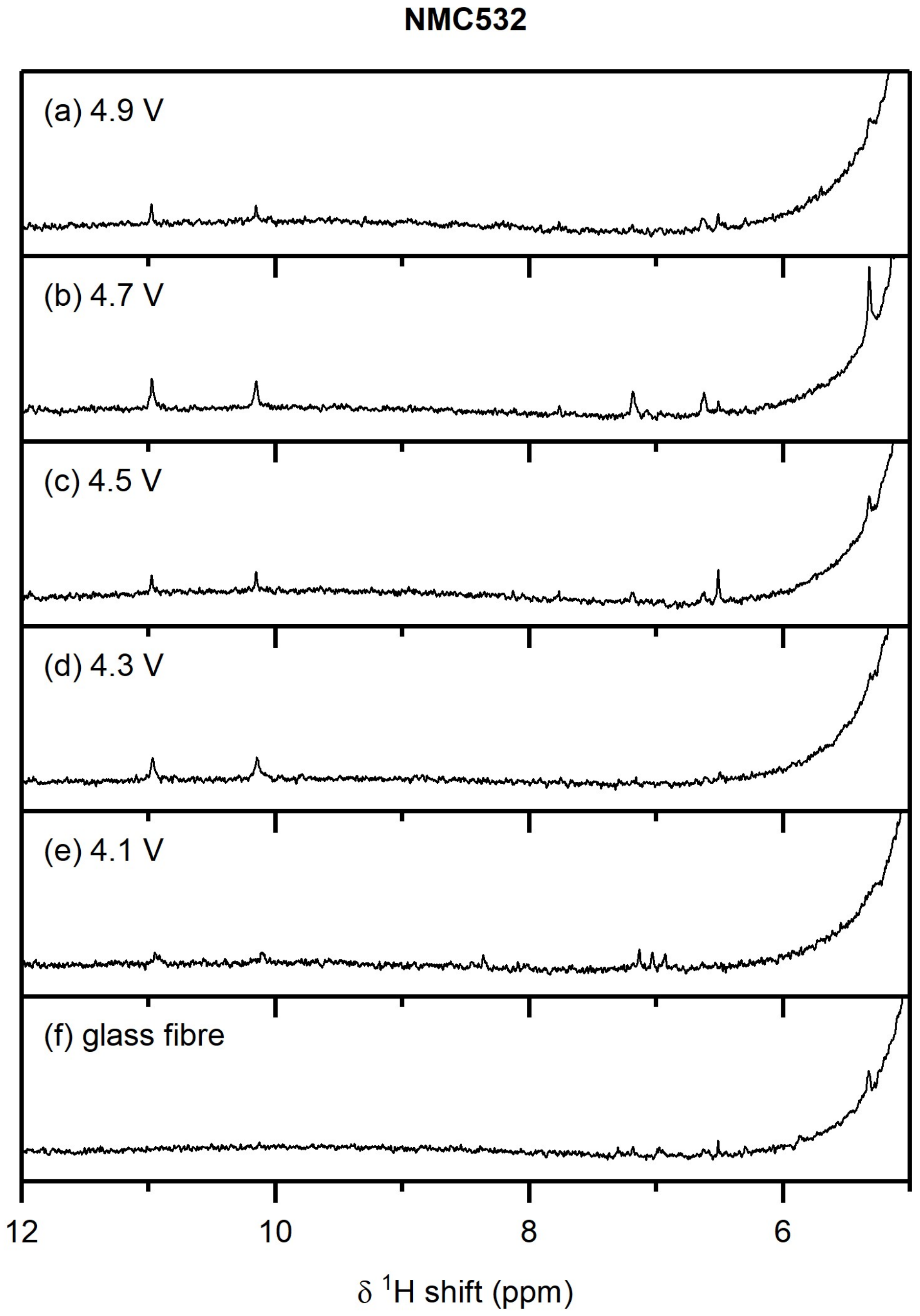

Figure S16. ^{1}H NMR spectra of electrolyte solutions extracted from NMC532/LFP cells after 10 cycles, where the cell cut-off voltages were chosen so the NMC electrode was cycled between (a) 4.9 V, (b) 4.7 V, (c) 4.5 V, (d) 4.3 V, (e) 4.1 V and 3.0 V vs Li^{+}/Li, and (f) pristine electrolyte solution. The cells were cycled at rate of C/5 in constant current-constant voltage (CCCV) mode.

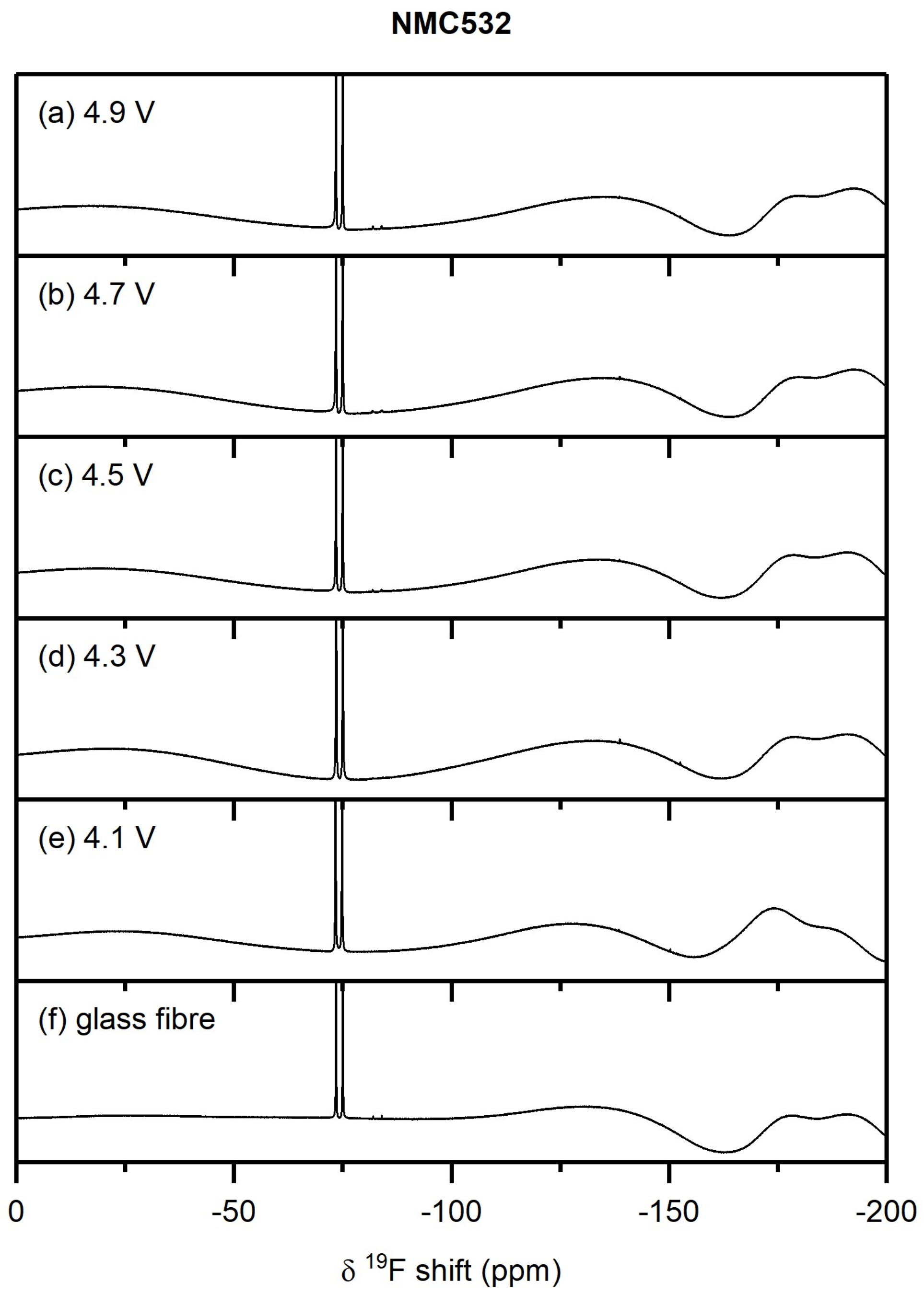

Figure S17. ^{19}F NMR spectra of electrolyte solutions extracted from NMC532/LFP cells after 10 cycles, where the cell cut-off voltages were chosen so the NMC electrode was cycled between (a) 4.9 V, (b) 4.7 V, (c) 4.5 V, (d) 4.3 V, (e) 4.1 V and 3.0 V vs Li^+/Li, and (f) pristine electrolyte solution. The cells were cycled at rate of C/5 in constant current-constant voltage (CCCV) mode.

6.5. NMC111/delithiated LFP cells

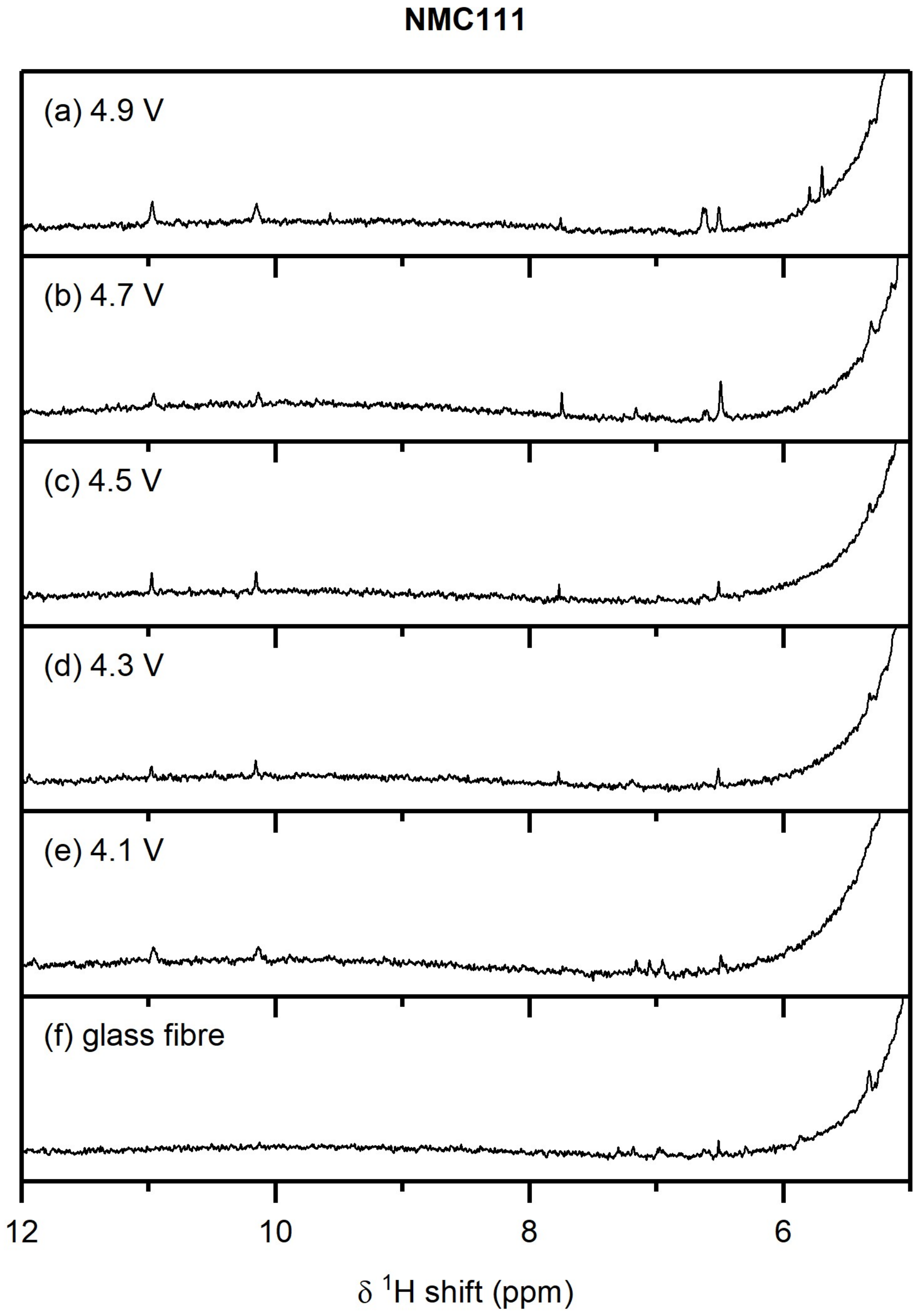

Figure S18. ^{1}H NMR spectra of electrolyte solutions extracted from NMC111/LFP cells after 10 cycles, where the cell cut-off voltages were chosen so the NMC electrode was cycled between (a) 4.9 V, (b) 4.7 V, (c) 4.5 V, (d) 4.3 V, (e) 4.1 V and 3.0 V vs Li^+/Li, and (f) pristine electrolyte solution. The cells were cycled at rate of C/5 in constant current-constant voltage (CCCV) mode.

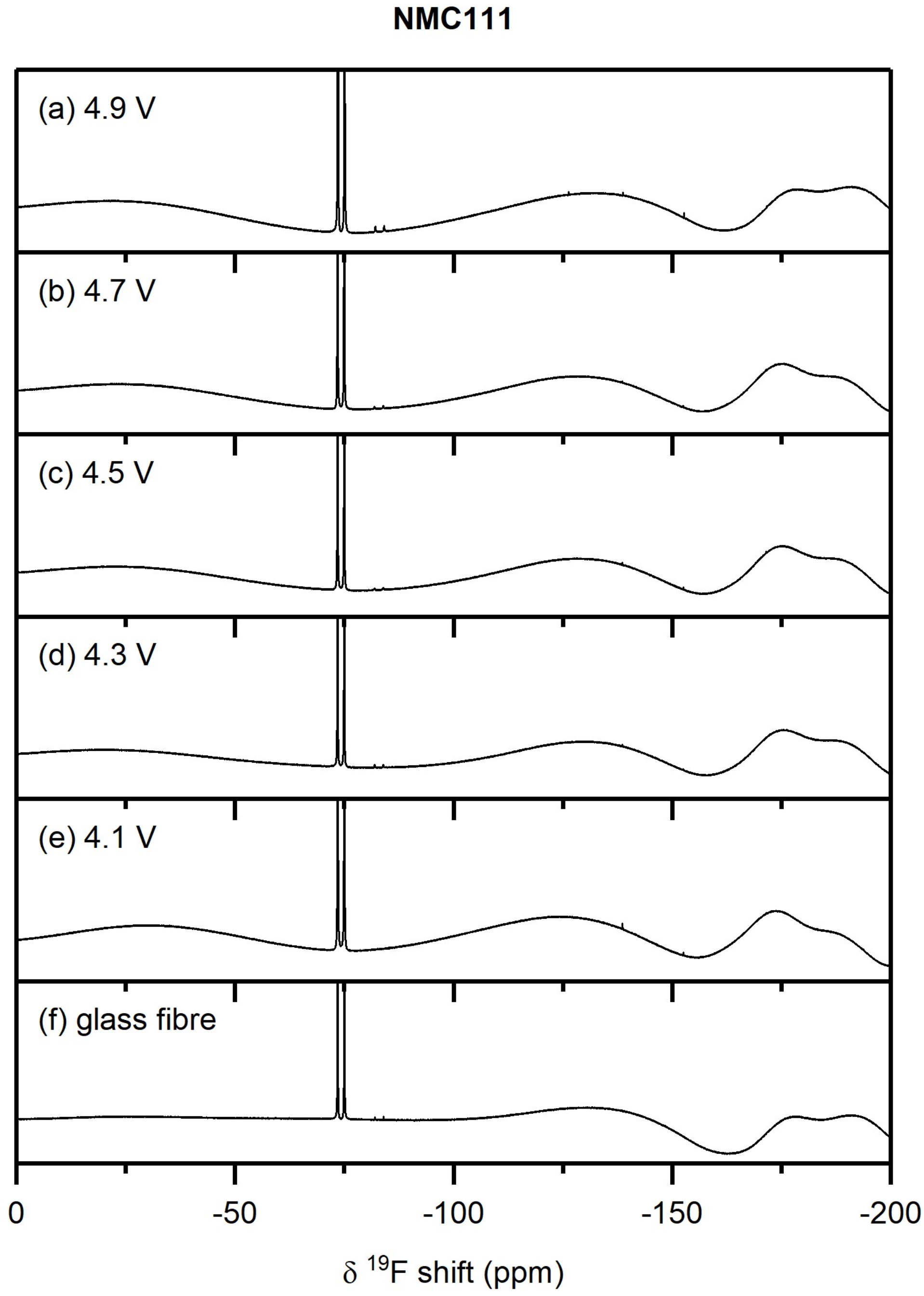

Figure S19. ^{19}F NMR spectra of electrolyte solutions extracted from NMC111/LFP cells after 10 cycles, where the cell cut-off voltages were chosen so the NMC electrode was cycled between (a) 4.9 V, (b) 4.7 V, (c) 4.5 V, (d) 4.3 V, (e) 4.1 V and 3.0 V vs Li^+/Li, and (f) pristine electrolyte solution. The cells were cycled at rate of C/5 in constant current-constant voltage (CCCV) mode.

6.6. LCO/delithiated LFP cells

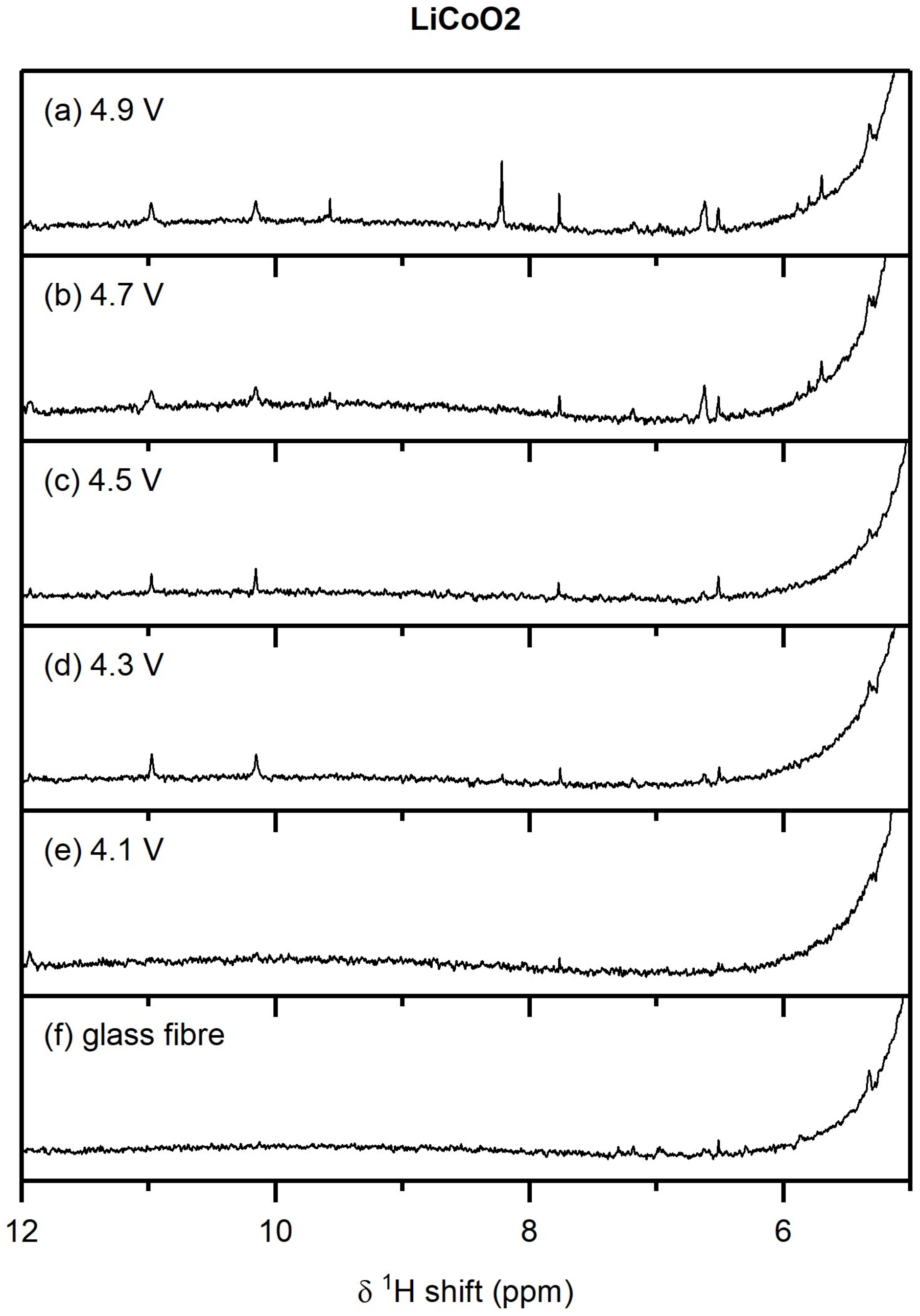

Figure S20. ^{1}H NMR spectra of electrolyte solutions extracted from LCO/LFP cells after 10 cycles, where the cell cut-off voltages were chosen so the NMC electrode was cycled between (a) 4.9 V, (b) 4.7 V, (c) 4.5 V, (d) 4.3 V, (e) 4.1 V and 3.0 V vs Li^{+}/Li, and (f) pristine electrolyte solution. The cells were cycled at rate of C/5 in constant current-constant voltage (CCCV) mode.

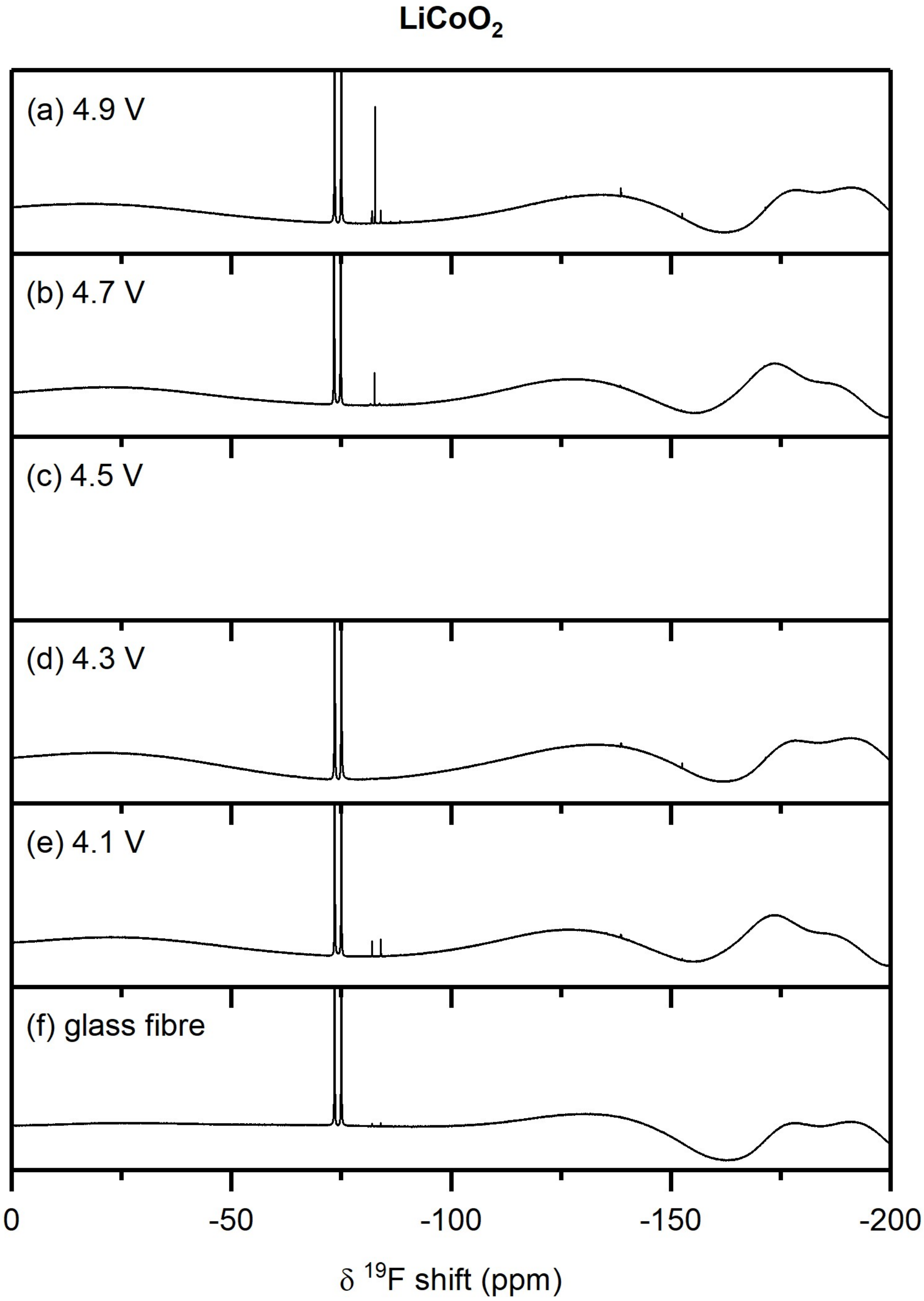

Figure S21. ^{19}F NMR spectra of electrolyte solutions extracted from LCO/LFP cells after 10 cycles, where the cell cut-off voltages were chosen so the NMC electrode was cycled between (a) 4.9 V, (b) 4.7 V, (c) 4.5 V, (d) 4.3 V, (e) 4.1 V and 3.0 V vs Li^+/Li, and (f) pristine electrolyte solution. The cells were cycled at rate of C/5 in constant current-constant voltage (CCCV) mode.

6.7. NMC/graphite and LCO/graphite cells

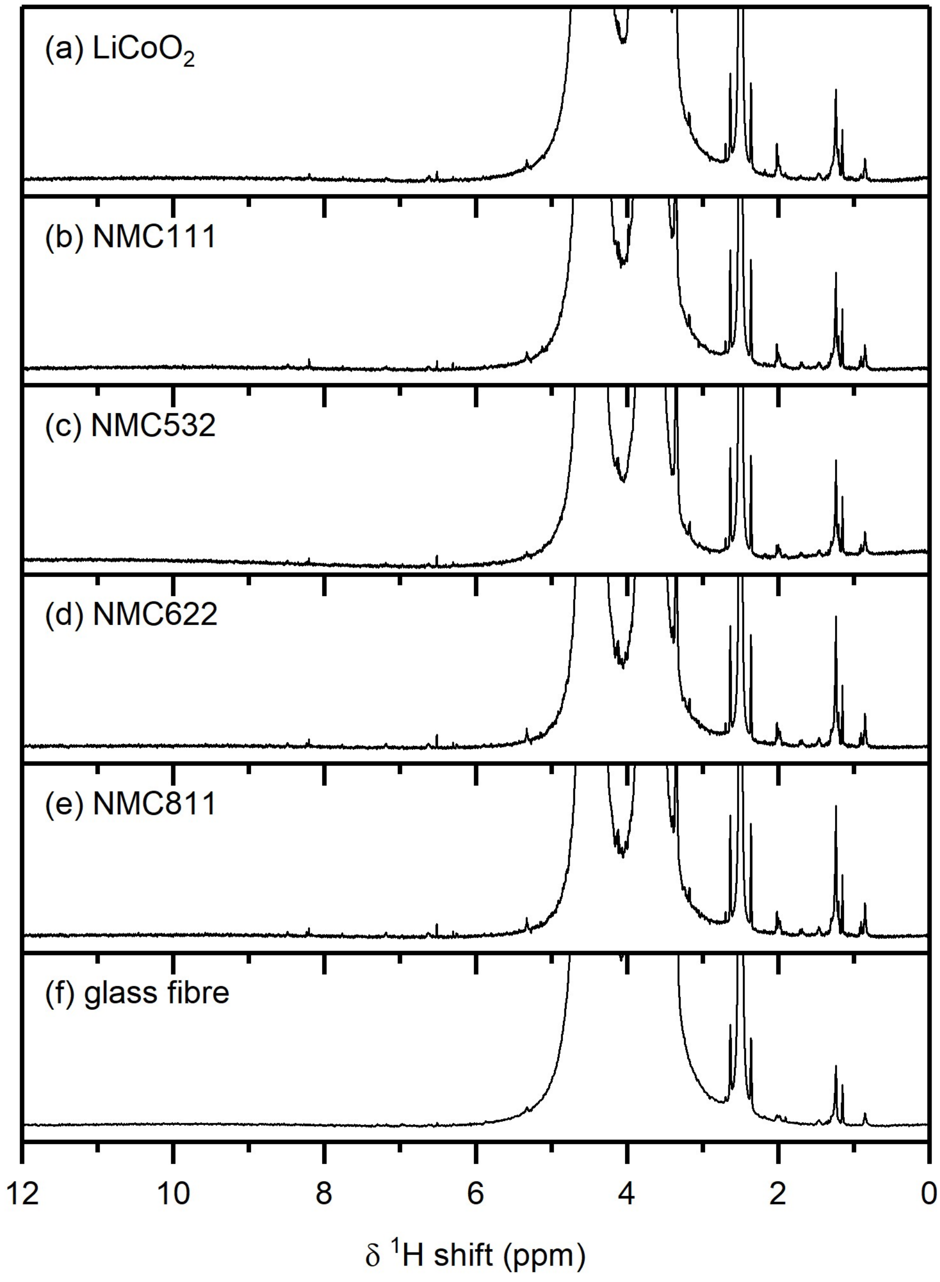

Figure S22. ^{1}H NMR spectra of electrolyte solutions extracted from (a) LCO/graphite, (b) NMC111/graphite, (c) NMC532/graphite, (d) NMC622/graphite and (e) NMC811/graphite cells after 10 cycles. The cells were cycled between 4.65–2.5 V_{cell}, corresponding to a V_{NMC} = 4.7 V and a $V_{graphite}$ = 0.05 V vs Li^+/Li, at rate of C/5 in constant current (CC) mode with a 2-hour potential hold at the top of charge.

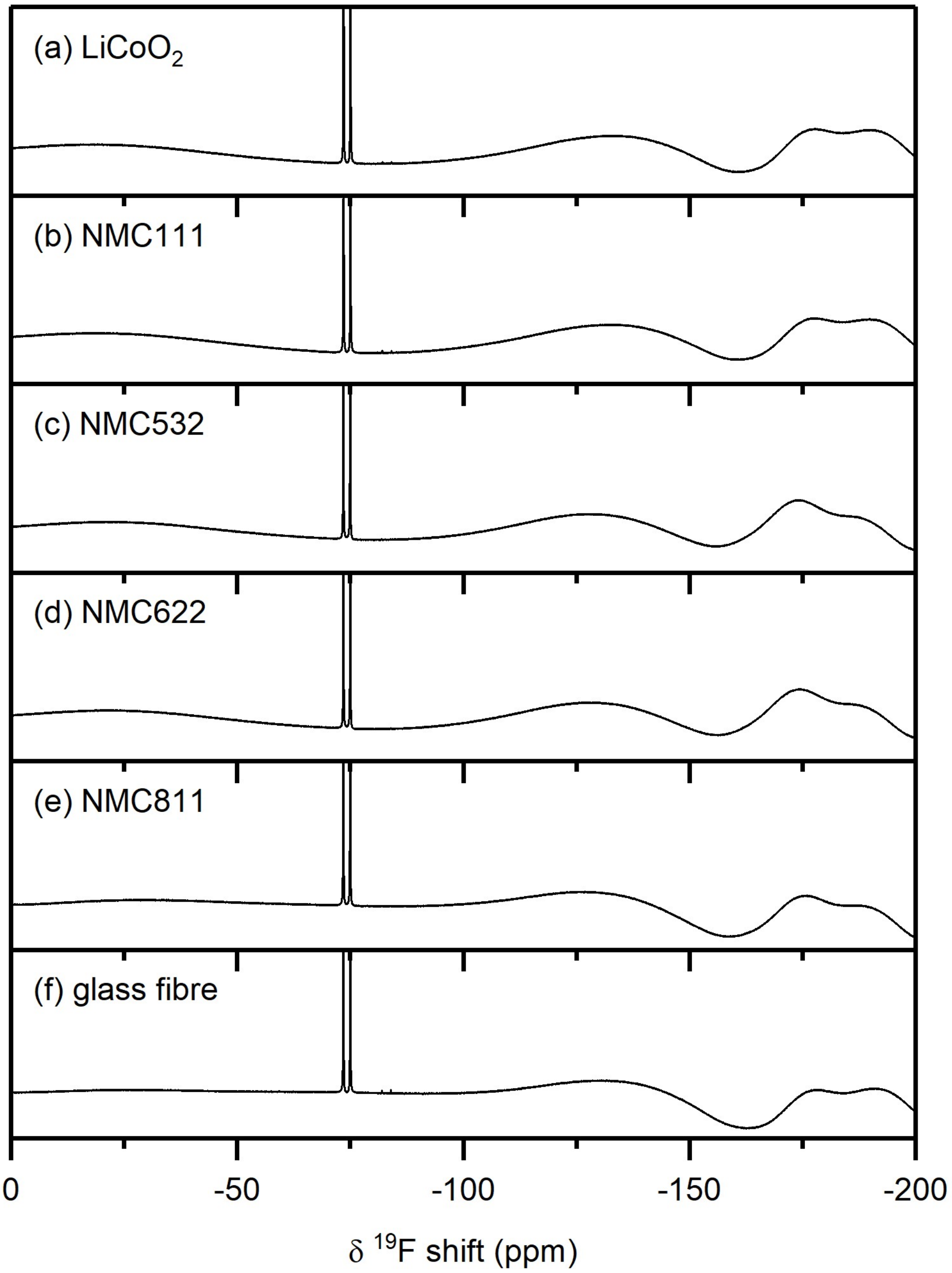

Figure S23. ^{19}F NMR spectra of electrolyte solutions extracted from (a) LCO/graphite, (b) NMC111/graphite, (c) NMC532/graphite, (d) NMC622/graphite and (e) NMC811/graphite cells after 10 cycles. The cells were cycled between 4.65–2.5 V_{cell}, corresponding to a V_{NMC} = 4.7 V and a $V_{graphite}$ = 0.05 V vs Li^+/Li, at rate of C/5 in constant current (CC) mode with a 2-hour potential hold at the top of charge.

6.8. Reactions between singlet oxygen and the carbonate solvent

To confirm that $^1O_2$ is produced, 9,10-dimethylanthracene (DMA; 99%, Sigma-Aldrich) is added (30 mM) to a 100 µM Rose Bengal (RB) EC solution, and the solution is irradiated with light at 525 nm for 10 min. DMA selectively reacts with $^1O_2$ to form an endoperoxide and does not react with $^3O_2$ or other reactive oxygen species (superoxide, peroxide, etc.). After irradiating the solution, 0.1 mL was taken for analysis by solution NMR and a shift of the signals from 8.34 and 7.56 ppm (pristine) to 7.48 and 7.33 ppm (after irradiation) in the ^1H spectrum indicates the formation of the endoperoxide species.[1]

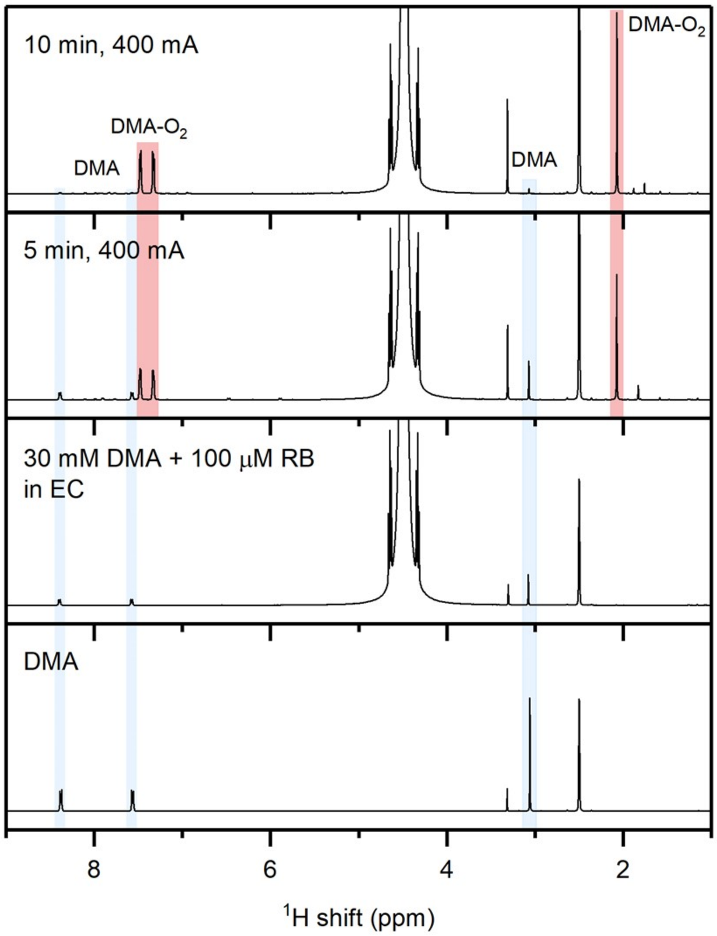

Figure S24. ^1H NMR spectra of an ethylene carbonate (EC), Rose Bengal (100 µM), and 9,10-dimethylanthracene (DMA, 30 mM) solution, after (a) 10 minutes, (b) 5 minutes and (c) before irradiation at 525 nm to generate singlet oxygen ($^1O_2$). (d) The spectrum of pure DMA in DMSO-d_6. After 5 minutes, the presence of DMA-O_2 was detected, indicating the formation of $^1O_2$ in the solution.

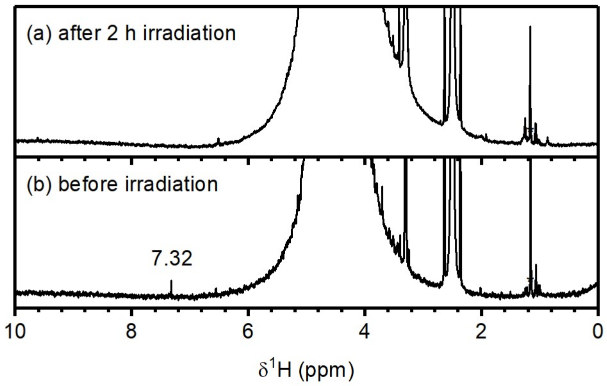

Figure S25. The expanded ^{1}H NMR spectra of those shown in Figure 9: ethylene carbonate (EC) and Rose Bengal (100 µM) solution (a) after and (b) before 2 hours of irradiation at 525 nm to generate singlet oxygen ($^{1}O_2$). No vinylene carbonate (VC, δ ^{1}H = 7.77 ppm) was detected. The signal arising from the Rose Bengal dye disappeared after irradiation, presumably due to photodecomposition reactions.

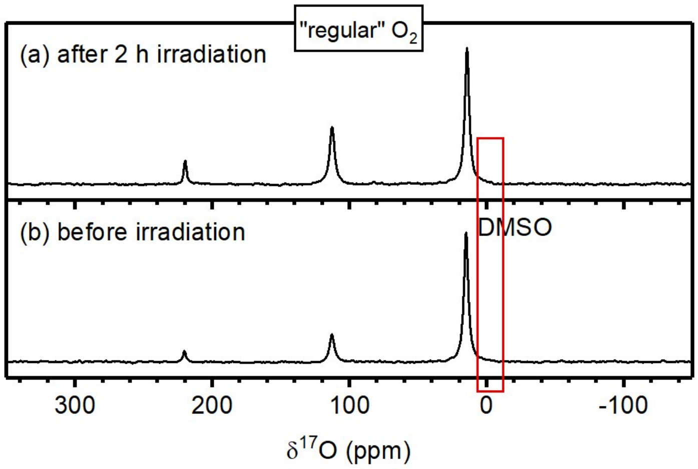

Figure S26. ^{17}O NMR spectra of an ethylene carbonate (EC) and Rose Bengal 100 µM) solution (a) after and (b) before 2 hours of irradiation at 525 nm to generate singlet oxygen ($^{1}O_2$). No new signals appeared after generating $^{1}O_2$ in solution.

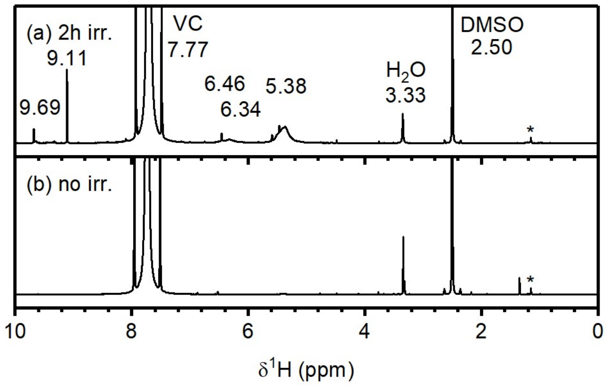

Figure S27. ^{1}H NMR spectra of a vinylene carbonate (VC) and Rose Bengal (100 µM) solution (a) after and (b) before 2 hours of irradiation at 525 nm to generate singlet oxygen ($^{1}O_2$). The chemical shifts of VC, H2O, DMSO and the reaction products are annotated in black.

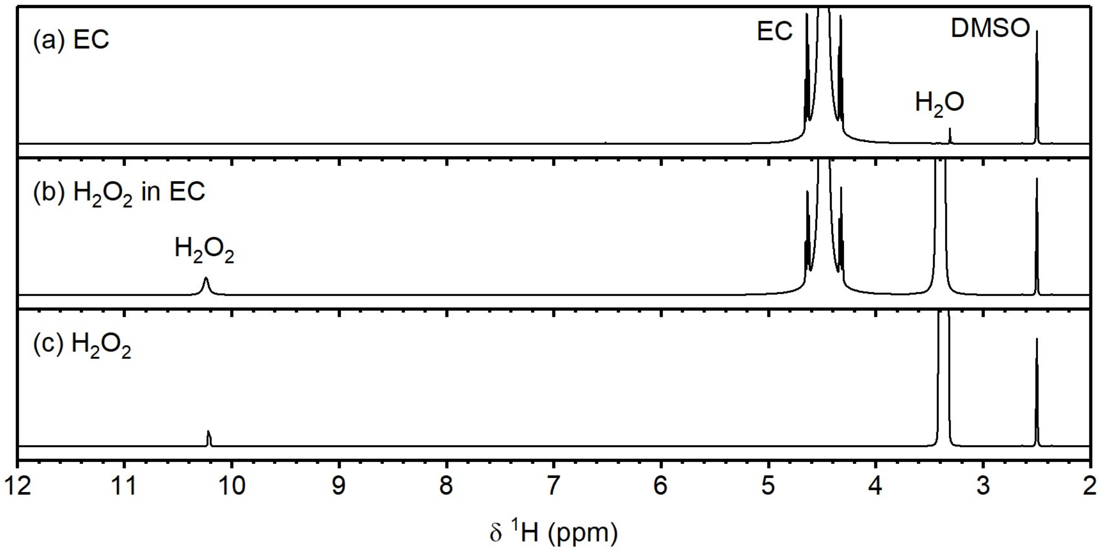

Figure S28. ^{1}H NMR spectra of (a) ethylene carbonate, EC; (b) 5000 ppm H_2O_2 in EC; (c) H_2O_2. DMSO-d_6 is used as the deuterated NMR solvent. The ^{1}H NMR spectrum of H_2O_2 in EC (b) shows that the lifetime of H_2O_2 in EC is long enough to observe a signal for H_2O_2

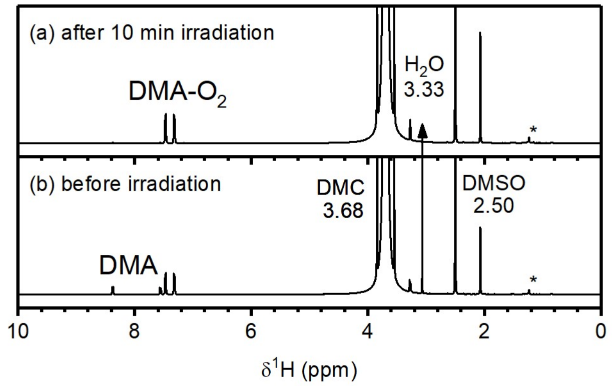

Figure S29. ^{1}H NMR spectra of a dimethyl carbonate (DMC), Rose Bengal (100 μM) and 9,10-dimethylanthracene (DMA, 30 mM) solution (a) after and (b) before 10 minutes of irradiation at 525 nm to generate singlet oxygen ($^1O_2$). The chemical shifts of DMC, H_2O and DMSO are annotated in black.

6.9. Assignment of the ^{1}H NMR signal at 3.98 ppm

The incorrect assignment of the ^{1}H NMR signal at 3.92 ppm in previous work by some of the authors[2] was discovered after comparison with a ^{1}H NMR spectrum of glycolic acid in DMSO-d_6 (Figure S30). The peak at 3.98 ppm is now assigned to the fluorophosphate ester $OPF_2(OCH_3)$, based on the appearance of a signal at 3.98 ppm in the ^{1}H NMR spectra of LP30 with 2 vol.% methanol added (Figure 10 in main text).

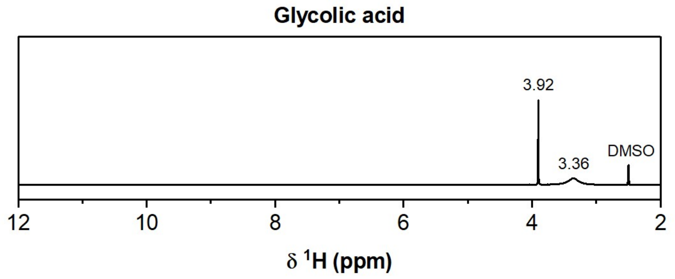

Figure S30. ^{1}H NMR spectrum of glycolic acid in DMSO-d_6; the ^{1}H NMR signals are observed at 3.92 ppm (s) and 3.36 ppm (broad), the peaks being assigned to the CH_2 and -OH groups, respectively.

7. References

(1) Mahne, N.; Renfrew, S. E.; McCloskey, B. D.; Freunberger, S. A. Electrochemical Oxidation of Lithium Carbonate Generates Singlet Oxygen. *Angew. Chemie - Int. Ed.* **2018**, *57* (19), 5529–5533. https://doi.org/10.1002/anie.201802277.

(2) Rinkel, B. L. D.; Hall, D. S.; Temprano, I.; Grey, C. P. Electrolyte Oxidation Pathways in Lithium-Ion Batteries. *J. Am. Chem. Soc.* **2020**, *142* (35), 15058–15074. https://doi.org/10.1021/jacs.0c06363.
